# StrucNS reveals interaction-weighted network topology as the driving predictor of absolute stability of natural and de novo proteins

**DOI:** 10.1093/bioinformatics/btag718

**Published:** 2026-09-30

**Authors:** Archita Mullick, Prodromos Daoutidis, Benjamin J Hackel

**Affiliations:** Chemical Engineering and Materials Science, University of Minnesota, Minneapolis, MN 55455, United States; Chemical Engineering and Materials Science, University of Minnesota, Minneapolis, MN 55455, United States; Chemical Engineering and Materials Science, University of Minnesota, Minneapolis, MN 55455, United States

## Abstract

**Motivation:**

Folded protein function requires stability, yet mapping structure and sequence to a fitness landscape remains difficult. The protein fold is the physical realization of complex, spatially-sensitive physicochemical interactions; quantitatively elucidating how these subtle relationships dictate thermodynamic stability remains challenging. We present StrucNS, a mathematical framework that identifies principles governing protein fitness by employing network science to learn physicochemical relationships between residues and their stability contributions directly from the protein fold. While the fold is traditionally viewed as the phenotypic consequence of the underlying chemical forces, we represent the fold as a network topology to decode the physicochemical dependencies that govern protein stability. Unlike language models reliant on high-dimensional evolutionary embeddings, StrucNS extracts these signals directly from the interaction-weighted network topology. Evolutionary independence uniquely suits StrucNS for de novo design prediction.

**Results:**

Despite reduced dimensionality and training depth, StrucNS outperforms unsupervised ESM-2 and ProteinMPNN on predicting mutational stability across three miniprotein test sets. StrucNS performs comparably to supervised UniRep in predicting absolute stability of de novo designs and outperforms supervised UniRep and supervised ESM-2 in predicting mutational stability of multi-mutants far outside its training regime. Feature analysis reveals network topology drive predictive power. SHAP analysis reveals high degree and low modularity of polar/hydrophobic mixed subnetworks as influential features, which highlights connectivity between the hydrophobic core and protein surface as predictive of stability contrary to the conventional focus on the hydrophobic core. Revelation of predictive topological features underscores the utility of interpretability.

**Availability:**

Source codes and datasets: https://github.com/Hackel-Group-CEMS/StrucNS and https://zenodo.org/records/20497907.

## 1 Introduction

Proteins represent the ultimate frontier of programmable matter, serving as the primary functional scaffolds that orchestrate the complexity of biological life (Anfinsen 1973, Dobson 2003). From the precision of intracellular signal transduction to the robust mechanics of systemic immunity, the utility of these macromolecules is derived from their ability to adopt unique three-dimensional architectures (Jumper *et al*. 2021). Beyond their endogenous roles, the capacity to rewrite protein sequences to provide improved or unique function is the cornerstone of modern biotechnology, enabling the synthesis of targeted therapeutics, high-sensitivity diagnostics, and industrial biocatalysts (Marks *et al*. 2011, Huang *et al*. 2016). At the heart of these advancements lies the physical requirement, for folded proteins, that a linear polypeptide sequence must not only fold into a specific geometry but also possess the thermodynamic stability necessary to resist the entropic drive toward unfolding (Fersht 1999, Dill and MacCallum 2012) all while enabling functionality (e.g. binding or catalysis). Understanding the determinants of this stability empowers protein engineering and is a prerequisite for interpreting the functional consequences of natural genetic variation (Tokuriki and Tawfik 2009).

Despite decades of research, the protein fitness landscape remains a *terra incognita* of immense dimensionality. The spatial geometry of a fold is the physical manifestation of many-body physicochemical interactions—a complex interplay of hydrophobic packing, hydrogen-bonding networks, and configurational entropy (Dill 1990, Levy and Onuchic 2004). However, the precise mapping between 3D coordinates and the underlying energy landscape remains an enduring enigma of molecular biophysics. Traditional experimental paradigms, such as directed evolution, attempt to traverse this landscape via stochastic sampling (Arnold 2018). While powerful, these methods are fundamentally constrained by the “curse of dimensionality,” as they can only explore a small fraction of the potential $20^{N}$ sequence space—a space larger than the number of atoms in the observable universe (Levinthal 1969). Conversely, rational design seeks to apply structural priors to introduce targeted mutations, yet our fragmentary understanding of the non-linear dependencies and the subtle topological constraints and geometric tensions within the native state often results in unreliable heuristics, limiting the success of classical force-field modeling (Alford *et al*. 2017).

As an alternative approach, machine learning has emerged as a transformative framework for inferring the protein energy landscape. Modern deep-learning architectures, including protein language models and graph neural networks, have demonstrated an unprecedented ability to learn the statistical regularities inherent in vast biological datasets, acting as high-throughput digital surrogates for select laboratory assays (Ingraham *et al*. 2019, Meier *et al*. 2021). However, as these models evolve in complexity, a fundamental interpretability crisis has emerged: a trade-off between predictive accuracy and physical transparency (Reichstein *et al*. 2019, Sethi and Zhou 2024). While these black-box architectures achieve high empirical accuracy, they offer little insight into the structural logic governing their predictions. For instance, in the design of *de novo* proteins, which lack the evolutionary history that modern models often rely upon, an opaque model may correctly flag a candidate as unstable without identifying the specific topological defect responsible (Listov *et al*. 2022). This lack of mechanistic feedback creates a bottleneck in the design cycle; without knowing *why* a fold fails, the engineer cannot inform the next round of sampling, effectively stalling the discovery pipeline. Consequently, there is a pressing need for a framework that transcends pattern matching to recover the fundamental structural rules that drive protein stability.

A dichotomy currently exists in protein modeling between zero-shot inference and high-capacity supervised learning. Zero-shot models have fundamentally redefined our ability to navigate sequence space by treating proteins as a structured language, learning the “evolutionary grammar” from vast genomic repositories (Alley *et al*. 2019, Rives *et al*. 2021). By distilling statistical patterns of conservation and co-variation, sequence-centric models—most notably protein language models (PLMs) such as ESM-2—capture a global summary of biological plausibility, enabling the prediction of mutational effects across diverse protein families without stability-specific training (Meier *et al*. 2021, Lin *et al*. 2023). Within this paradigm, the emergence of ProteinMPNN has established a new benchmark for structure-based zero-shot design (Dauparas *et al*. 2022). By utilizing a deep message-passing neural network to learn the conditional probability of an amino acid sequence given a fixed three-dimensional backbone, ProteinMPNN effectively captures the local packing requirements and steric constraints of the native state (Ingraham *et al*. 2019). However, despite its generative prowess, ProteinMPNN is primarily optimized for the inverse folding problem; it identifies sequences that are *compatible* with a fold but does not explicitly quantify the energetic magnitude of thermodynamic stability or the biophysical drivers of destabilization.

To achieve the high-resolution precision required for biochemical applications, researchers have increasingly turned to one-shot and supervised learning frameworks. In these paradigms, the high-dimensional latent representations of PLMs, such as the 1280-dimensional embeddings of ESM-2 or the 1900-dimensional hidden states of UniRep, are recycled as informative priors for specialized downstream tasks (Biswas *et al*. 2018). For instance, UniRep embeddings have been successfully utilized to guide the optimization of green fluorescent proteins, TEM-1 β-lactamase and industrial enzymes with minimal experimental data (Alley *et al*. 2019, Biswas *et al*. 2021). Building on this logic, ThermoMPNN and the SPURS (Stability Prediction Using a Rewired Strategy) framework represent the state-of-the-art in integrating structural and evolutionary signals (Dieckhaus *et al*. 2024, Li and Luo 2025). ThermoMPNN combines the structural encoding of ProteinMPNN with a supervised regressor trained on the Megascale dataset (Tsuboyama *et al*. 2023) of 272 712 variants to map structural features directly to changes in the Gibbs free energy of unfolding (ΔΔG) (Dieckhaus *et al*. 2024). SPURS further pushes this synergy by “rewiring” the structural features from ProteinMPNN into the attention layers of ESM-2, effectively conditioning evolutionary signals on 3D geometric constraints to predict absolute stability (Li and Luo 2025). The impact of these architectures extends beyond benchmark accuracy to experimentally validated design: ProteinMPNN redesign of myoglobin and TEV protease produced variants with improved expression, elevated melting temperatures, and, for TEV protease, catalytic activity exceeding both the parent sequence and previously reported engineered variants (Sumida *et al*. 2024), while ESM-2 alone generates de novo sequences that fold, with 152 of 228 experimentally characterized designs proving soluble and monomeric (Verkuil *et al*. 2022). Across both prediction and design, these architectures constitute the prevailing paradigm in computational protein engineering.

Despite the power of these synergistic frameworks, a critical generalization ceiling and interpretability gap remain. While PLMs like ESM-2 and UniRep are powerful, their heavy reliance on evolutionary depth makes them prone to failure when applied to *de novo* proteins or orphan sequences that lack a rich history of natural selection (Hsu *et al*. 2022, Listov *et al*. 2022). Recent studies have highlighted the limitations of sequence-only models in navigating the “fitness deserts” of synthetic proteins, where the rules of biological plausibility are not yet established by evolution (Garcia *et al*. 2026). Furthermore, the reliance on dense latent embeddings creates a strategic bottleneck in the design cycle. Because these architectures operate within an opaque mathematical manifold, they lack the physical transparency required for informed engineering (Rudin 2019). When a black-box model rejects a candidate library, it offers no actionable mechanistic feedback (Adebayo *et al*. 2018, Ghorbani *et al*. 2019). Post-hoc explanation methods like SHAP (SHapley Additive exPlanations) have been shown to be fragile in these settings, with embedding-based attributions often failing to correspond to discrete biophysical entities such as salt bridges or hydrogen-bond networks. This lack of mechanistic grounding creates a trust gap in rational engineering. Consequently, there is a necessity for a framework that does not merely match patterns in a latent space but recovers the fundamental, interpretable structural rules that drive protein stability.

We propose that this bridge can be built through a network topology-based abstraction: conceptualizing the protein not as merely a fold geometry, but as an interaction-weighted network topology. This shift addresses a central challenge in structural biology: the realization that protein fold is the physical manifestation of hidden, multi-scale interactions (Barabási and Oltvai 2004, Sengupta and Kundu 2012). By applying this perspective at the Ångström scale, we can treat thermodynamic stability as an emergent property of the network topology, allowing us to test a fundamental hypothesis: that defined topological invariants can serve as sufficient surrogates for the intricate forces governing the fitness landscape (Estrada 2002).

To investigate this, we present StrucNS, a framework designed to decode the physicochemical logic of stability of a fold through a topological lens. Unlike existing models that rely on high-dimensional evolutionary embeddings (Jumper *et al*. 2021, Lin *et al*. 2023), StrucNS operates as a “no-embedding” framework, deriving its predictive power directly from the explicit connectivity of a residue interaction network (RIN) (Amitai *et al*. 2004, Piovesan *et al*. 2016, Brysbaert and Lensink 2021). The first pillar of our approach focuses on the hierarchical organization of the fold. We partition the protein into discrete topological subnetworks based on chemical environments, such as the hydrophobic core and polar surface, to analyze the balance between connectivity within a community and the interactions across these boundaries. This allows us to determine whether the predictive signal for stability can be recovered directly from the topological organization of these communities, offering a pathway to evaluate *de novo* protein variants where evolutionary history, the primary signal for current machine learning models, is absent or poorly defined (AlQuraishi 2021, Dauparas *et al*. 2022).

The residue interaction network is itself a mature representation, but its use in structural biology has been predominantly analytical rather than predictive. Centrality measures computed on RINs identify functionally important residues (Amitai *et al*. 2004), characterize protein–protein binding interfaces (Brysbaert and Lensink 2021), and trace allosteric communication through energy-weighted network formalisms (Vijayabaskar and Vishveshwara 2010). Where RINs have addressed stability directly, the treatment has been largely descriptive, characterizing the network signatures of stable folds rather than predicting stability for unseen variants (Brinda and Vishveshwara 2005). Community detection on protein structure networks has likewise been applied mechanistically, linking modular reorganization of residue communities to allosteric transitions (Stetz and Verkhivker 2017). Supervised structure-based stability predictors have meanwhile converged on graph neural architectures that learn latent node and edge representations over k-nearest-neighbour residue graphs (Dauparas *et al*. 2022, Dieckhaus *et al*. 2024, Li and Luo 2025), achieving high accuracy at the cost of representations that are not expressed in terms of discrete physicochemical interactions. The closest precedent to the present framework is NeEMO (Giollo *et al*. 2014), which computes a RIN for a wild-type structure, extracts centrality and environment descriptors for a mutated position, and predicts ΔΔG, establishing that RIN-derived features carry genuine stability signal. NeEMO addresses a different problem, however: it estimates the relative energetic consequence of a single substitution from the local environment surrounding the mutated residue, whereas StrucNS predicts absolute thermodynamic stability from the global organization of the fold, including for multi-site variants and de novo designs in which no wild-type reference exists. Energy-weighted RIN construction and modularity-based partitioning are established techniques, and StrucNS builds on both; its departure lies in what is subsequently done with the partitioned network. Force-based representations of proteins are likewise well established, as elastic network models treat residues as beads connected by harmonic springs to characterize fluctuations and collective motions about the native state, retaining physical coordinates throughout (Tirion 1996, Bahar *et al*. 1997, Atilgan *et al*. 2001). Force-directed layout algorithms such as Fruchterman–Reingold (Fruchterman and Reingold 1991) originate instead in graph drawing and serve principally for visualization. StrucNS applies force relaxation for neither purpose. We impose a hierarchical spring balance in which attractive forces scale with physicochemical interaction weight and repulsive forces act between non-interacting residues, resolving the network to a topological equilibrium that constitutes a new coordinate system rather than a depiction of the existing one. Coupled with modularity-based community detection (Blondel *et al*. 2008) and a multi-label physicochemical labeling scheme that assigns communities to overlapping functional subnetworks across hydrophobicity, charge, and flexibility axes, this yields an intermediate representation distinct from both the input RIN and the native fold. It is from this object, rather than from the RIN itself, that predictive signal is extracted.

The descriptors computed from this representation—centrality, modularity, spread, edge density, and related quantities—are standard network science measures. Their value lies downstream: because each corresponds to an explicit property of a defined subnetwork, feature attribution returns statements about protein architecture rather than about latent dimensions. Three elements therefore distinguish this framework from prior work: the transformation of an energy-weighted RIN into an interaction-weighted network topology through hierarchical force equilibrium; the labeling of communities into overlapping functional subnetworks, together with the inter-subnetwork features this enables; and the resulting capacity for mechanistic interpretability applied to absolute stability across natural and de novo sequences.

To validate the generalizability of this topological logic, we benchmarked StrucNS against diverse mutational datasets, spanning both natural folds and synthetic designs. StrucNS outperforms unsupervised PLMs in predicting mutational stability, reveals the value of a network science perspective on modeling protein structure/stability relationships, and highlights an interaction-weighted network topology as a key determinant of protein stability prediction. StrucNS outperforms unsupervised ESM and MPNN models in predicting mutational stability across three miniprotein sets, especially in de novo folds. StrucNS performs similarly to supervised UniRep and is competitive with supervised ESM-2 despite reduced model dimensionality and training depth. Crucially, despite being trained exclusively on miniproteins, StrucNS demonstrates generalizability, achieving performance on large globular proteins over 10 times the size of the folds in the training set.

StrucNS offers a transparent, biophysically grounded lens through which to understand and engineer the absolute stability of both natural and de novo proteins. Our analysis reveals that network topology is the primary driver of protein stability, as evidenced by 59% feature importance and 53% recall reduction upon topology feature ablation. Intriguingly, our findings reveal that the hybrid polar/hydrophobic subnetworks are more influential than the hydrophobic core in determining protein stability, with high connectivity and low modularity as stabilizing features. By unifying these topological insights with high-throughput stability prediction, StrucNS demonstrates that the precise architecture of energy-weighted residue interactions is a fundamental determinant of protein fitness, providing a powerful and interpretable framework for the computational design of robust, highly stable proteins.

## 2 Methods

### 2.1 StrucNS architecture

We hypothesize that thermodynamic stability, in both natural and de novo proteins, is an emergent property of hierarchical interaction topology. To test this, we developed StrucNS, a multi-scale framework that decodes high-dimensional spatial data into interpretable topological signals. For clarity, we define the following nomenclature: the protein fold refers to the explicit three-dimensional spatial coordinates of the residues; network topology represents the protein as an abstract graph, defined by its nodes (residues), edges (interactions), and spatial positioning.

StrucNS (Fig. 1) operates through network science operations: (i) construction of an energy-weighted RIN from in-silico structures (Jumper *et al*. 2021, Wu *et al*. 2022); (ii) identification of mesoscopic partitions of dense interactions through community detection; (iii) identification of macroscopic subnetworks through functional semantic labeling; (iv) mechanical equilibrium of an interaction-weighted network; (v) calculation of network features; and (vi) model training.

**Figure 1:**
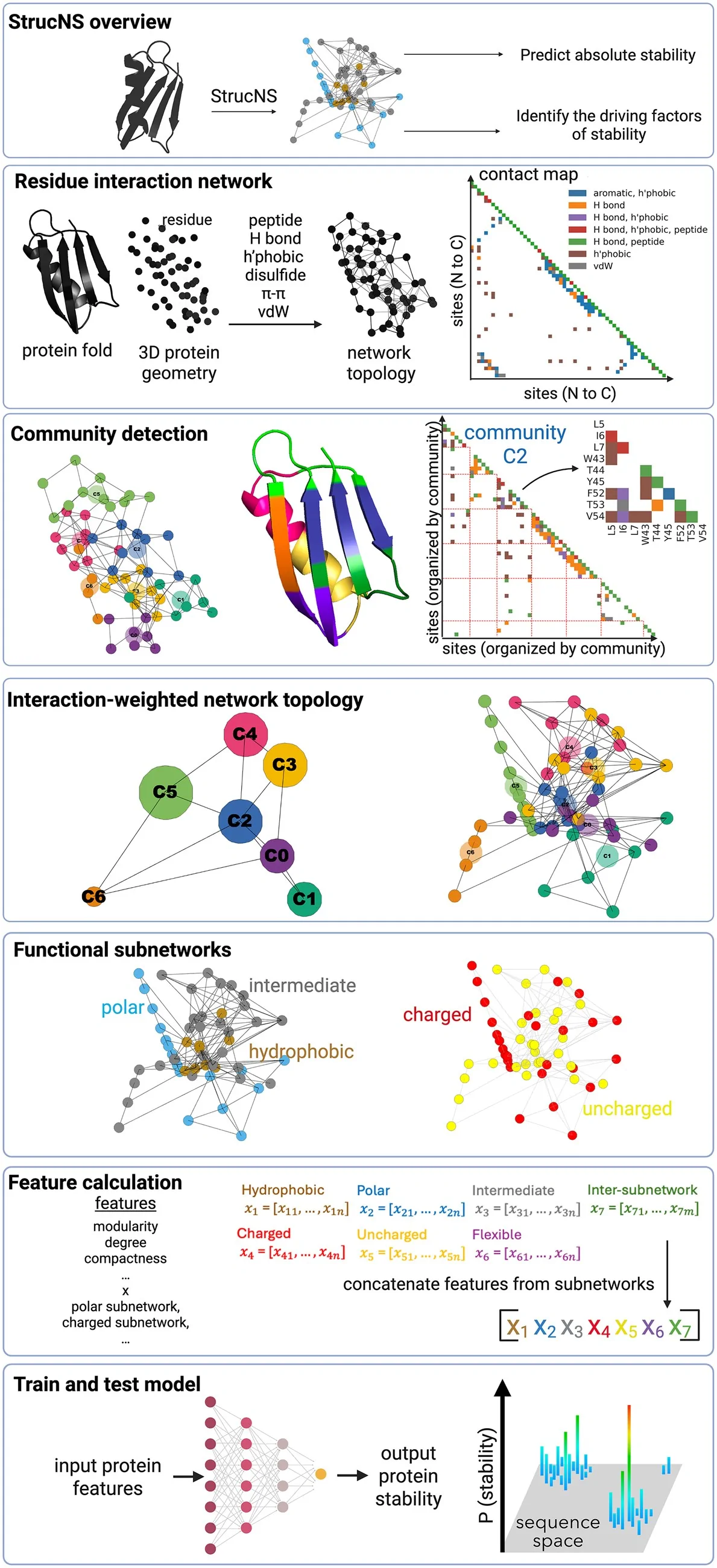
Overview of StrucNS model. (*StrucNS overview.*) StrucNS uses protein folds to build residue interaction networks and community-driven network topology, allowing it to predict absolute thermodynamic stability while feature analysis enables determination of the driving factors of protein stability. A ββαββ protein (PDB: 1PGA) is used to exemplify the StrucNS approach. (*Residue interaction network (RIN).*) The three-dimensional protein fold is converted into a discrete RIN in which each amino acid centroid is a node and six bond types are identified as edges based on spatial and physicochemical criteria. These interactions are shown as a contact map, with white being not connected and color being a type of interaction. (*Community detection.*) Communities are then detected as clusters of nodes based on modularity maximization. As shown in the community contact map, these well-connected clusters appear as dense blocks along the diagonal, while the rest of the matrix remains sparse, highlighting the modular nature of the identified communities. Community 2 (C2) is enlarged as an example to show its members (residues 5, 6, 7, 43, 44, 45, 52, 53, 54). (*Interaction-weighted network topology projection.*) Node positions are then adjusted by performing a spring balance with attractive edges and repulsive non-edges to generate an interaction-weighted network topology projection. (*Functional subnetworks.*) Communities are then labeled and grouped into functional subnetworks on the basis of physicochemical composition (polar, intermediate, hydrophobic; charged or uncharged). (*Features calculation.*) The labeled topology is then evaluated for a series of network science features within and across subnetworks. (*Train and test model.*) This collection of features is then used as inputs to train a mathematical model to predict protein stability as the output across a broad swath of protein structures and sequences. The resultant model enables a map of the sequence-stability landscape.

#### 2.1.1 Residue interaction network (RIN)

Amino acid sequences were converted to FASTA format and processed using OmegaFold (v1.1.0). Structure predictions were performed using the 60M-parameter model with default weights and four cycles. Computations were executed on NVIDIA A100 GPUs with a 15-minute runtime limit per sequence. We modeled the protein tertiary structure as an energy-weighted RIN using the Biopython (v1.81) PDB parser and NetworkX (v3.1). Nodes in the network represent residue centroids, serving as the effective centers of interaction mass for each side chain (Fig. 1). Edges represent specific physicochemical interactions: covalent peptide bonds, hydrogen bonds, hydrophobic contacts, disulfide bridges, aromatic stacking, and van der Waals forces. We established these edges using stringent atomic distance thresholds, such as a 3.5Å limit for hydrogen bonds between donor and acceptor atoms of neighboring residues. Detailed criteria are provided in SI Appendix, S1.1, available as supplementary data at *Bioinformatics* online. To reflect disparate thermodynamic contributions, we weighted edges using established bond dissociation energies and experimental stability scales (SI Appendix, S1.2, available as supplementary data at *Bioinformatics* online). Figure 1  *RIN* illustrates the protein’s tertiary structure and network topology, including residue coordinates and edge weights. The resulting connectivity map highlights the network’s sparseness and the diverse interaction types between residues.

#### 2.1.2 Community detection via weighted modularity optimization

The RIN provides a high-resolution map of atomic interactions, yet the resulting topology is inherently high-dimensional, as seen in the contact map (Fig. 1, *RIN*). To mitigate structural noise common in structure prediction models like OmegaFold (Wu *et al*. 2022), we transitioned from a single-node representation to a coarse-grained, mesoscopic framework. We applied a network partitioning strategy based on modularity maximization to identify discrete community structures (Fig. 1, *Community detection*). In this context, we hypothesized that because RIN edges and weights reflect physical chemical interactions, modularity optimization would isolate “interaction-dense” communities. These clusters represent residue groups where the internal density of biochemical bonds significantly exceeds the connectivity expected from a random null model. This null model serves as a critical statistical baseline, representing stochastic connectivity while preserving the degree of each residue (SI Appendix, S1.3, available as supplementary data at *Bioinformatics* online). Using the Louvain algorithm (Blondel *et al*. 2008) on the energy-weighted RIN, we decomposed *N* protein residues into *K* discrete structural modules (K<N). Notably, these communities frequently incorporate residues distant in primary sequence. For instance, Community 2 (C2) spans disparate secondary structures, including multiple β-sheets (Fig. 1  *Community detection*). This partitioning remains entirely network-driven and agnostic to specific residue identities. These communities, marked by triangles along the contact map diagonal, exhibit higher internal connectivity than interactions with the rest of the network.

#### 2.1.3 Interaction-weighted network topology via spring balance

Protein tertiary structure arises from the interplay between short-range packing forces within a secondary structure and long-range interactions across secondary structures. To encode this information, we transformed the protein fold into an interaction-weighted network topology by treating the network topology as a collection of springs. Connected nodes experienced an attractive force proportional to the edge weight, while unconnected nodes experienced a repulsive force proportional to the inverse of 1/4th of their distance. We applied a force balance (SI Appendix, S1.4, available as supplementary data at *Bioinformatics* online) on the nodes using the Fruchterman-Reingold algorithm (Fruchterman and Reingold 1991) to reach a topological equilibrium (Fig. 1, *Interaction-weighted network topology projection*). This new projection represents a state of topological equilibrium that embeds interactions into the projection space. First, we applied a spring-balance to this global topology to rearrange nodes and communities according to equilibrium. Next, we applied spring-balance to the intra-community nodes until they reached equilibrium. We performed this in two steps based on the hypothesis of resolving local and global level forces. Models in literature, such as AlphaFold3 (Abramson *et al*. 2024), have achieved success by resolving local and global scale interactions, which informed our choice of this hypothesis.

#### 2.1.4 Macroscopic subnetworks through functional semantic labelling

Modularity optimization partitions the protein into structurally coherent units based on interaction density. However, these emergent communities are initially defined by arbitrary numerical indices that lack biochemical context. To bridge mathematical interaction-weighted network topology with the thermodynamic principles governing protein stability, we implemented multi-functional semantic labeling (SI Appendix, S1.5, available as supplementary data at *Bioinformatics* online) to identify functional subnetworks (Fig. 1, *Functional subnetworks*). While community detection identifies clusters of densely interconnected residues, semantic labeling defines the “biochemical personality” of these modules. This process characterizes how each functional subnetwork contributes to the global stability of the protein. We hypothesize that communities are biophysically pleiotropic; a single community may exert multiple, simultaneous functions on the fold. For instance, a rigid hydrophobic community may concurrently harbor charged residues at its solvent-exposed surface, fulfilling dual roles. To capture this complexity, we labeled each community using a composite hydrophobicity metric (derived from normalized Kyte-Doolittle (Kyte and Doolittle 1982) and Grantham scales (Grantham 1974)), charge metrics, and flexibility thresholds (SI Appendix, S1.6, available as supplementary data at *Bioinformatics* online). We grouped communities with shared labels into three primary subnetworks: hydrophobic, polar, and intermediate (Fig. 1, *Functional subnetworks*). For example, Community 4 (C4) belongs to both the intermediate (grey) and charged (red) subnetworks, and Community 3 (C3) resides within both the hydrophobic (brown) and uncharged (yellow) subnetworks.

#### 2.1.5 Functional subnetwork-based and inter-subnetwork feature extraction

By this stage, we have established an interaction-weighted network topology partitioned into overlapping functional subnetworks. We now translate this architecture into a feature space, defined as a vector of parameters describing network science features of individual functional subnetworks (e.g. modularity of each polar, hydrophobic, etc.) and inter-subnetworks (e.g. boundary flux between polar and hydrophobic) (full feature list and descriptions in SI Appendix, S1.7, available as supplementary data at *Bioinformatics* online). These features derive three types of information: connectivity-based, protein fold geometry-based, and interaction-weighted network topology-based. Connectivity-based features depend solely on nodes and edges, including modularity, the density of internal connections relative to a random null model, edge density, the ratio of actual edges to all possible connections, and degree, the number of connections per residue. Fold geometry-based features depend on the distribution of residues in 3D space. Interaction-weighted network topology-based features inform the interaction-weighted geometry of subnetworks, such as the spatial clustering coefficient of each subnetwork, which quantifies the local node density within the interaction-weighted space. Once we estimate these features from each of the six subnetworks and the inter-subnetwork features, we concatenate them into a single vector to form the feature space of the protein (Fig. 1, *Feature calculation*).

### 2.2 Training and test sets

To construct a robust predictive framework, we utilized a dataset with absolute protein stability measured, via cDNA display proteolysis, for 844 425 natural and de novo proteins (Tsuboyama *et al*. 2023). The Megascale dataset was downloaded from Zenodo (https://zenodo.org/records/7992926). We filtered the primary dataset (Tsuboyama2023_Dataset1_20230416.csv) to retain datapoints which had confidence interval lower than ∼3 kcal/mol to ensure that the target labels, representing the experimental change in Gibbs free energy, originated from high-quality thermodynamic measurements. To rigorously evaluate whether our model learns universal physicochemical principles or simply memorizes specific backbone geometries, the data were partitioned into three mutually exclusive test sets based on protein family identity and mutational coverage (Fig. 2A). The data were divided such that 80% of the available variants from 80% of parental folds were used for model training. The remaining 20% of variants from those same parental folds were set aside as Test Set 1 (TS1). This internal validation set measures the model’s ability to interpolate within known structural architectures by predicting the stability of unseen mutational variants on scaffolds that were already presented during the optimization phase. To evaluate true structural generalization, we established two additional external holdout sets, Test Set 2 (TS2) and Test Set 3 (TS3), which contain protein families that were entirely excluded from the training process. These sets represent unseen folds that the model has never encountered during its optimization. TS2 comprises de novo designed proteins—synthetic structures engineered by researchers to adopt specific folds, while TS3 consists of naturally occurring parental folds. Success on TS2 and TS3 would demonstrate a capacity to generalize structural logic across the structural universe and prove that the model can interpret the stability of entirely novel architectures rather than relying on the specific geometric features of the training examples.

**Figure 2:**
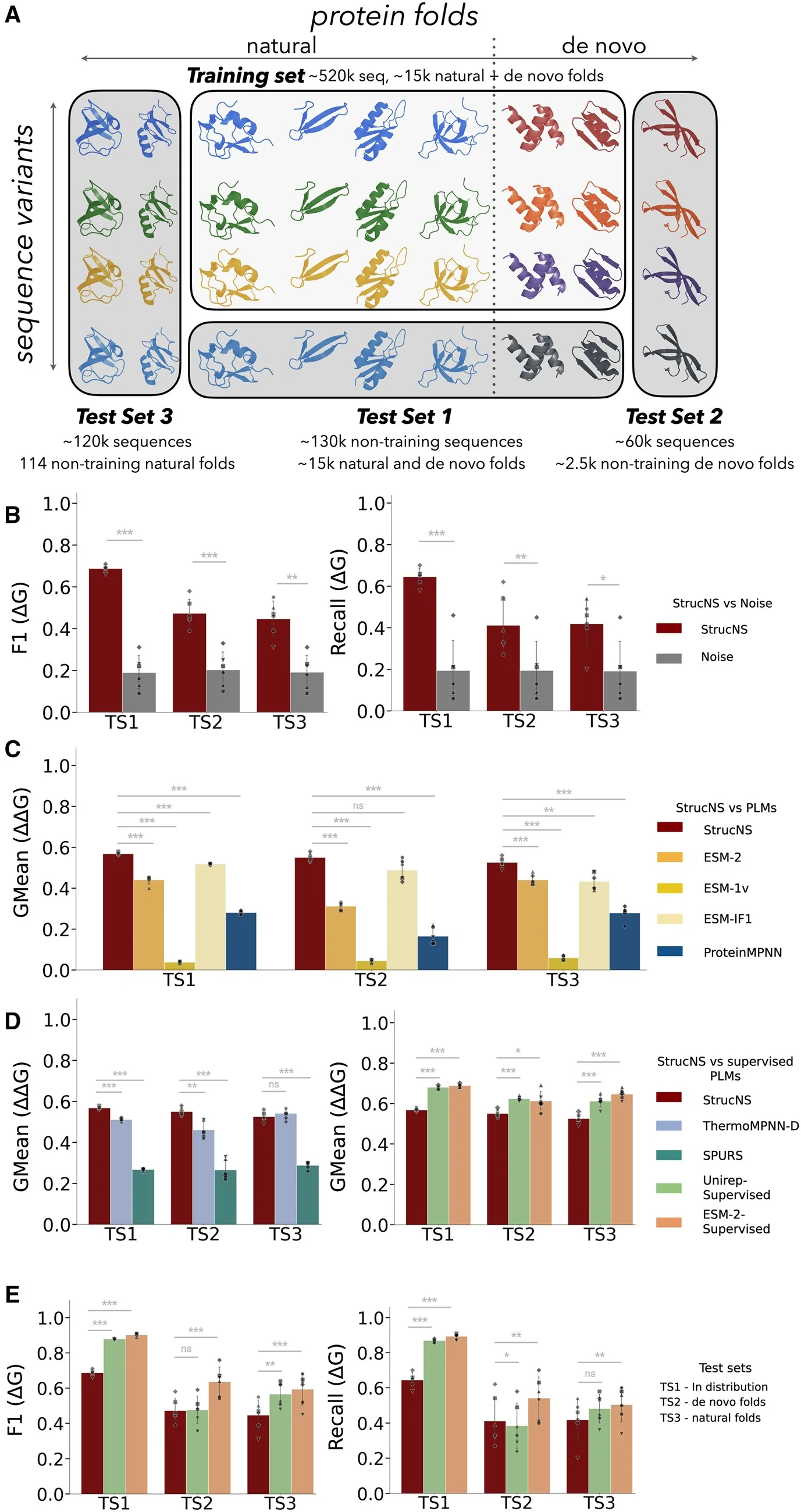
The predictive performance of StrucNS across broad test sets (A) was evaluated in comparison to (B) statistical noise, (C) evolutionary models, and (D, E) high-capacity embeddings in supervised protein language models. StrucNS was trained on 80% of sequences within 80% of protein folds within the 8 × 10^5^ sequence-stability measurements in the Megascale data set (Tsuboyama *et al*. 2023). (A) Data are presented for evaluation across three test sets: TS1: sequences held out of training from parental folds included in model training; TS2: de novo designed proteins with parental folds excluded from training; TS3: natural protein folds excluded from training. Data are shown for each of six independent train/test splits (symbols) along with the mean and standard deviation. Statistical significance relative to StrucNS—as computed via paired t-test—is depicted with * (0.01 <  *P* ≤ 0.05), ** (0.001 <  *P* ≤ 0.01), and *** (*P* ≤ 0.001). (B) The F1 statistic (left) and recall (right) for predicting the stability classification (ΔG > 3 kcal/mol) of variants is shown for StrucNS and a noised model in which feature values were randomly permuted across all variants, thereby preserving the feature distribution but ablating connection to the proper stability output. (C) The geometric mean of classwise sensitivity (GMean) for predicting the proper change (increase or decrease) in the probability of stability upon mutation was computed using StrucNS or protein language models ESM-2 (Lin *et al*. 2023), ESM-1v (Meier *et al*. 2021), ESM-IF1 (Hsu *et al*. 2022), or ProteinMPNN (Dauparas *et al*. 2022). (D) The GMean for predicting mutational stabilization was computed using StrucNS, ThermoMPNN (Dieckhaus *et al*. 2024), and SPURS (Li and Luo 2025) (left), which was trained on the same Megascale data set, and supervised implementations of UniRep or ESM-2 (right). (E) Recall and F1 for predicting the stability classification of variants was computed for StrucNS and the supervised protein language models.

### 2.3 Performance metrics

The predictive objective was formulated as a binary classification task to distinguish robustly stable protein variants from unstable or marginally stable backgrounds. While thermodynamic stability is conventionally bounded at a free energy of unfolding, ΔG>0, we imposed a stricter classification threshold of approximately two hydrogen bonds (Pace *et al*. 2014) , ΔG>3 kcal/mol. This rigorous cutoff was selected to isolate variants with high structural resilience, effectively filtering out neutral drift and ensuring that “stable” classifications correspond to designs with significant thermodynamic utility. The 95% confidence interval on Δ G within the Megascale dataset is 0.17 kcal/mol, although the distribution extends to 2.2 kcal/mol at the 99${\;}^{th}$ percentile. Thus, our selected threshold of ΔG>3 kcal/mol ensures stability. We evaluated the impact of this threshold and demonstrate that ΔG>3 kcal/mol is an informative threshold that does not bias StrucNS performance (SI Appendix, S3.1, available as supplementary data at *Bioinformatics* online). In protein engineering workflows, downstream experimental characterization represents the primary bottleneck; therefore, we assessed precision to minimize the resource burden of validating false positives. Recall was simultaneously monitored to ensure the computational filter maintained sufficient coverage of the fitness landscape, preventing the exclusion of diverse, viable candidates. We use the harmonic mean of these two metrics, F1, as a key merged metric. To further evaluate performance in the context of mutational stability, we employed the geometric mean (GMean) of sensitivity and specificity, a metric that provides a balanced assessment of classification accuracy across imbalanced datasets. The StrucNS model generates a likelihood of stability (ΔG>3 kcal/mol), from which we derived a log-likelihood ratio (LLR) to compare each variant against the wild-type (WT) sequence. Variants with an LLR>0.05 were classified as stabilizing mutations, while those with LLR<−0.05 were categorized as destabilizing. (SI Appendix, S3.2, available as supplementary data at *Bioinformatics* online). By utilizing GMean as a primary benchmark for mutational stability, we ensured a robust comparison of our approach against existing computational models.

### 2.4 Machine learning model

To predict stability, we developed a binary classification model where samples were labeled as stable for ΔG>3 kcal/mol and unstable for ΔG≤3 kcal/mol. The architecture consists of a fully-connected feed-forward neural network implemented in TensorFlow, featuring a dynamic configuration of 1 to 5 hidden layers with widths ranging from 16 to 512 units. To ensure the robustness and generalization of our results, we generated six independent training/test cases, each utilizing a unique random seed to partition the full dataset into training, TS1, TS2, and TS3. This multi-split approach ensures that model performance is not an artifact of a specific data distribution. For each of these six cases, a dedicated model was developed through a rigorous hyperparameter optimization pipeline. Hyperparameter tuning for each case was conducted using the Optuna framework via 5-fold cross-validation. The predictive framework was implemented using TensorFlow (v2.12) and Keras. To optimize the neural network architecture, we utilized the Optuna (v3.2) framework, executing 300 trials managed via a SQLAlchemy persistent SQLite backend (optuna_study.db). The objective function targeted the maximization of the average peak $F_{1}$-score across all five folds (SI Appendix, S2, Fig. S2, available as supplementary data at *Bioinformatics* online). To maintain the integrity of the validation process, a StandardScaler was fitted strictly on the training folds and applied to the validation fold to prevent data leakage. We also implemented fold-wise splitting for 5-fold cross validation, ensuring all sequences belonging to the same fold were grouped within the same fold to avoid overestimating accuracy through sequence similarity. The optimization objective maximized the $F_{1}$ score using the optuna.trial interface to dynamically sample architectural parameters: number of hidden layers (1–5), layer units (suggest_categorical), Dropout rates (0.1–0.5), and learning_rate (logarithmic scale $10^{-4}$ to $10^{-1}$). Data preprocessing was handled through scikit-learn, employing StandardScaler to normalize feature distributions. To prevent data leakage, scaling parameters were computed strictly on training folds and applied to validation/test sets via the fit_transform and transform methods. Cross-validation was managed through sklearn.model_selection.KFold with 5 splits. Model performance was monitored using a custom keras.callbacks callback to track $F_{1}$ scores at each epoch end, while EarlyStopping was implemented to monitor val_loss with a patience of 8–10 epochs. The final model weights were selected based on the peak validation $F_{1}$ score while monitoring validation loss. Statistical evaluation was performed using sklearn.metrics to compute precision, recall, and confusion matrices. Final artifacts, including the trained model and normalization parameters, were serialized using final_model.save() (H5 format) and joblib.dump() (GZ compression).

## 3 Results

### 3.1 Model performance and comparative benchmarking

#### 3.1.1 Validation against statistical noise

To assess the ability of StrucNS to capture genuine biophysical signals rather than statistical artifacts of amino acid composition, we compared its stability predictions against a noise model. We generated this control by randomly permuting feature values across all variants, preserving the underlying feature distribution while scrambling the predictive signal. In absolute stability (ΔG) classification, the noise model failed to exceed an F1 score of 0.20, while StrucNS reached 0.69 (TS1), 0.47 (TS2), and 0.44 (TS3) (Fig. 2B). Divergence in recall was comparably stark as the noise model plateaued at ∼0.19, whereas StrucNS achieved robust recall (Mean TS1: 0.64; TS2: 0.40; TS3: 0.42; Fig. 2B). This performance gap demonstrates the efficacy of our network-based approach.

#### 3.1.2 Performance against large-scale PLMs

We next evaluated StrucNS against prominent PLMs, including ESM-2 (Lin *et al*. 2023), ESM-1v (Meier *et al*. 2021), and ESM-IF1 (Hsu *et al*. 2022) (Fig. 2C). ESM-2 is a general-purpose transformer; ESM-1v is optimized for zero-shot variant prediction; and ESM-IF1 is an inverse folding model conditioning sequences on structure. These models are trained on vast datasets, ESM-2 and 1v on up to 250 million sequences (UniRef) and ESM-IF1 on 12 million AlphaFold structures, with parameters ranging from 650 million to 15 billion. With only ∼ 520K sequences of training, StrucNS achieved superior GMean across all test sets, particularly in de novo folds (TS2) where StrucNS maintained a GMean of 0.55 while ESM-2 collapsed to 0.31. ESM-1v yielded the lowest performance (GMean < 0.07), supporting the hypothesis that sequence-based evolutionary likelihoods struggle when the wild-type lacks a rich evolutionary history. ESM-IF1 and ProteinMPNN (Dauparas *et al*. 2022) also trailed StrucNS, with ProteinMPNN dropping to 0.17 in TS2. Crucially, while TS2 and TS3 represent true out-of-distribution regimes for StrucNS, they are likely represented within the massive training corpora of the ESM suite and ProteinMPNN. Despite this data disadvantage and a significantly smaller parameter count, StrucNS demonstrates higher resilience in zero-shot prediction by identifying the broader topological relationships defining a stable fold. Details of ESM and ProteinMPNN model implementations are added in SI Appendix, S6.1, S6.2, S6.3, S6.4, available as supplementary data at *Bioinformatics* online.

#### 3.1.3 StrucNS vs ThermoMPNN-D and SPURS

We compared our network topology approach to ThermoMPNN-D (Dieckhaus *et al*. 2024) (SI Appendix, S6.5, available as supplementary data at *Bioinformatics* online), a graph neural network (GNN)-based architecture trained directly on the same MegaScale dataset used for StrucNS. We also evaluated performance against SPURS (Li and Luo 2025), a deep-learning framework that integrates sequence embeddings from ESM-2 with structural hidden features from ProteinMPNN, followed by dynamic graph rewiring and downstream GNN processing. For mutational impacts (ΔΔG), StrucNS outperformed ThermoMPNN-D and SPURS in both TS1 and TS2 (Fig. 2D) and outperformed SPURS in TS3. Notably, while TS2 and TS3 are truly out-of-distribution for StrucNS, these data points were part of the explicit training set and priors for ThermoMPNN-D and SPURS. Despite this, StrucNS matches or exceeds the performance of specialized GNN-based architectures.

#### 3.1.4 Comparison with supervised PLMs

Finally, we compared StrucNS to supervised models utilizing high-capacity UniRep (SI Appendix, S6.7, available as supplementary data at *Bioinformatics* online) and ESM-2 (SI Appendix, S6.6, available as supplementary data at *Bioinformatics* online) embeddings (Fig. 2E). These supervised PLMs encode both evolutionary information and stability patterns through dual-round training in a black box manner, representing the performance ceiling. The supervised ESM-2 and supervised UniRep baselines shown in Fig. 2D and 2E were trained on the same sequences under the same six family-aware train/test partitions used for StrucNS, with identical hold-out definitions for TS1, TS2, and TS3. In the de novo regime (TS2), StrucNS (F1: 0.47) matched UniRep (F1: 0.48) but was less effective than the billion-parameter ESM-2 (F1: 0.64). On natural folds (TS3), StrucNS (F1: 0.44) was competitive, albeit less effective, versus UniRep (F1: 0.51) and ESM-2 (F1: 0.55). TS2 and TS3 are truly out-of-distribution for StrucNS but likely present in the PLM pre-training corpora; our method achieves reasonable performance using explicit structural connectivity rather than massive latent embeddings.

#### 3.1.5 Prediction on single and multi-site mutations in large globular proteins

To evaluate the generalizability of StrucNS, we tested the model on standard single- and multi-site mutations of proteins that significantly exceed the size of the 40–72 amino acid miniproteins used during training. We utilized the PTMul (Montanucci *et al*. 2019, Dieckhaus *et al*. 2024 ) dataset for multi-site mutations and the KORPM (Hernández *et al*. 2023, Dieckhaus *et al*. 2024) and s669 (Pancotti *et al*. 2022, Li and Luo 2025) datasets for single-site mutations. PTMul contains multiple-site mutations derived from the ProTherm database, containing globular proteins featuring sizes typically ranging from 50 to over 500 residues. KORPM consists of single-point mutations curated from ProTherm and specifically curated to be balanced by removing an over-representation of alanine mutations and excluding data from extreme pH/temperature, covering a broad size distribution of 40–600 amino acids. The s669 dataset is a non-redundant set of mutations specifically filtered from ProTherm (non-overlapping with KORPM) and ensuring high-quality experimental ΔΔG values, with protein lengths spanning 50–450 residues. Notably, these datasets are entirely out-of-distribution for StrucNS, whereas a significant portion of their data was included in the training sets of the comparative models. As illustrated in Fig. 3, StrucNS outperforms all other models in GMean when predicting the likelihood of mutational stability for multi-site mutants. This indicates that despite being trained on miniproteins, StrucNS captures generalized topological and structural patterns that govern stability across diverse folds. In single-site mutant predictions, StrucNS performs similarly to ESM-2 and ESM-IF1, while slightly underperforming compared to ProteinMPNN, Unirep-S, and ESM-2-S. These results highlight the robustness of StrucNS across varying sizes and physicochemistry. We hypothesize that StrucNS excels in multi-site mutant prediction because it utilizes network topology to predict absolute stability, allowing it to discern complex topological differences between multi-mutants and wild-type structures. ProteinMPNN, the other structure-based model analyzed, demonstrated performance similar to StrucNS in these evaluations. The lack of superiority of StrucNS in single-site mutants may result from reduced structural and connectivity differences in single mutants.

**Figure 3:**
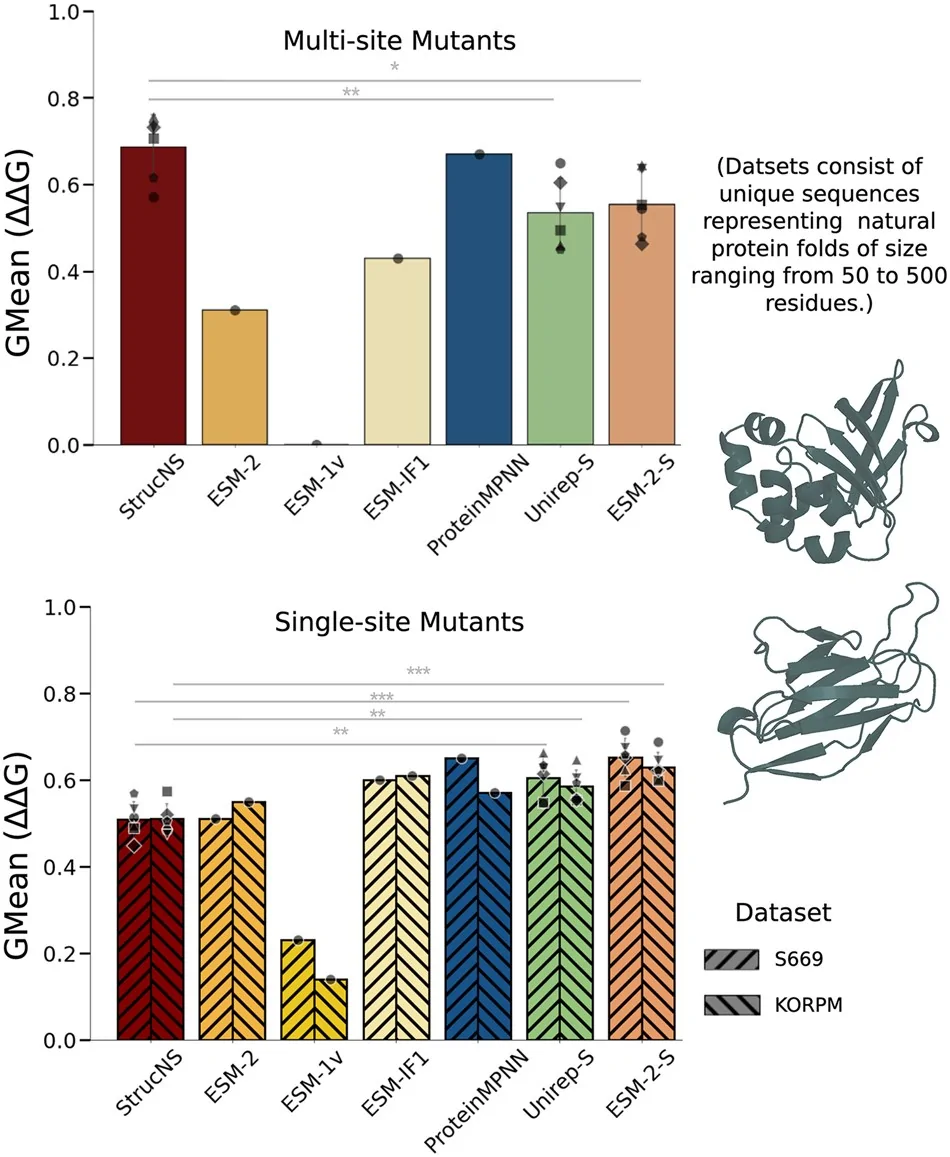
The predictive performance of StrucNS was evaluated in comparison to evolutionary models and high-capacity embeddings in PLMs. StrucNS was trained on miniproteins from the Megascale dataset (Tsuboyama *et al*. 2023). Data are presented for evaluations across the multi-site mutants dataset (top) and the single-site mutants dataset (bottom). Multi-site mutants dataset consists of curated data from literature having two or more mutations in proteins of size ranging from 50 to 500 residues and having large globular proteins. The single mutants dataset consists of datasets s669 and KORPM having single mutations and a size distribution of 40–600 residues. GMean for predicting mutational stabilization is shown.

### 3.2 Explainable AI reveals biophysical drivers of stability

Unlike black-box architectures, the StrucNS framework allows for a direct interpretation of the driving factors of protein stability. To deconstruct the biophysical drivers of stability, we utilized SHapley Additive exPlanations (SHAP) (Lundberg and Lee 2017) to quantify the additive contribution of each feature to the model’s prediction (Fig. 4 and SI Appendix, Fig. S11, available as supplementary data at *Bioinformatics* online). The primary driver, degree within intermediate subnetworks, shows that high connectivity within these hybrid polar/hydrophobic subnetworks correlates strongly with stability. The second most influential feature is inter-class fragment connectivity (ICFC) between intermediate and charged subnetworks, and the fifth is ICFC between intermediate and uncharged communities, while the fourth is low modularity in intermediate subnetworks. These four leading features suggest that it is beneficial for the intermediate subnetwork to have high connectivity and be integrated across the entire protein network rather than being isolated within itself to ensure stability. Aligned with these leading features, importance share analysis reveals that the intermediate community features are the most influential for stability prediction, even more than hydrophobic communities (Fig. 4B). While the hydrophobic core is traditionally viewed as the primary determinant of stability, these unexpected results suggest that the highly connected intermediate region, bridging the polar surface and the hydrophobic core, exerts a more significant influence. Furthermore, the high inter-class fragment connectivity (ICFC) highlights the critical role of connectivity between functionally distinct protein regions in maintaining structural integrity.

**Figure 4:**
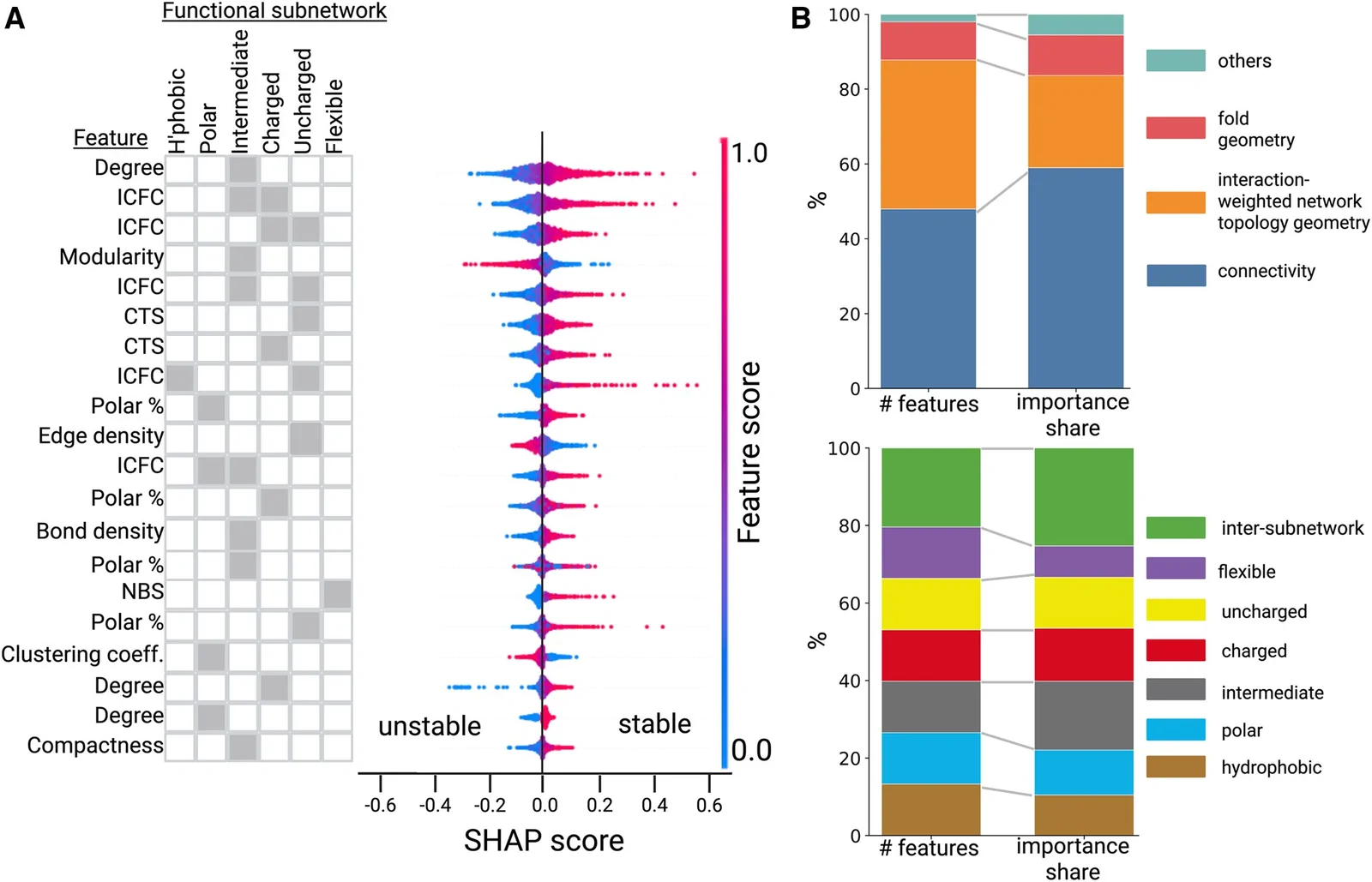
An explainable machine learning model reveals the topological determinants of protein stability. (A) *SHAP summary of biophysical drivers.* Individual topological features are ranked by their impact on model predictions using SHapley Additive exPlanations (SHAP). SHAP deconstructs a model’s decision-making by calculating the extent to which each feature shifts a specific prediction away from the baseline value (average model output across the training set; 0.25 likelihood of stability herein). Each point represents a protein variant. Its horizontal position on the *x*-axis indicates whether that feature’s value increased (positive SHAP) or decreased (negative SHAP) the prediction relative to the baseline. ICFC: inter-class fragment connectivity; CTS: class to community ratio. (B) (Top) *Feature Importance Share.* The importance share of predictive signal is aggregated into four model stages: connectivity-based, geometry-based, projection-based, and others. (Bottom) The importance share of predictive signal is shown for each subnetwork type. The number of features within each component is presented to enable comparison to the importance share.

More broadly, we computed the SHAP importance share for families of features as their fraction of the sum of absolute SHAP values across all features. Connectivity-based features—those computed purely from network—account for 59% of model importance, a significant rise from the 48% of such features (Fig. 4). Features that rely on the interaction-weighted network topology account for 28% of model impact. While influential, this share emerges from 117 features, 38% of all features; thus the relative impact per feature is much less than connectivity alone. Features computed from the original fold network topology contribute 10% from 10% of features. Details of implementation are provided in SI Appendix, S8, available as supplementary data at *Bioinformatics* online.

### 3.3 Model component analysis

#### 3.3.1 Network topology is more significant than fold geometry

To further evaluate the contributions of network topology versus geometry, we systematically introduced noise into each component and measured the performance of the ablated models. To isolate the predictive contribution of spatial coordinates from discrete graph features, we first performed a controlled geometry-noising experiment. Rather than introducing unconstrained structural disruption that could yield trivial nonphysical artifacts, we applied systematic perturbations to atomic coordinates using both random walk (SI Appendix, S7.2, available as supplementary data at *Bioinformatics* online) and random box (SI Appendix, S7.3, available as supplementary data at *Bioinformatics* online) coordinate displacement protocols. The random walk protocol generates a non-biological configuration by stepping along the chain while keeping each residue’s rigid shape intact, whereas the random box protocol uniformly scatters residue centroids throughout the native protein’s 3D bounding box to preserve global density while destroying sequential and structural context. These procedures selectively disrupt metric spatial distances and break the predictive signal arising directly from 3D coordinates while holding all secondary network representations constant. Specifically, the discrete residue interaction network, Louvain community assignments, and physicochemical subnetwork labels remain entirely preserved. By decoupling metric geometry from discrete network organization, this experiment directly evaluates whether model predictions depend on exact spatial atomic positions or the underlying relational topology. Upon the geometric noising (SI Appendix, S7.2, available as supplementary data at *Bioinformatics* online), the ablated model maintained performance levels comparable to StrucNS across all test sets, as evidenced by the minimal impact on F1 and recall (Fig. 5). A second version of geometric noising yielded equivalent results (SI Appendix, S7.3, available as supplementary data at *Bioinformatics* online). The results indicate that the precise spatial coordinates of the fold are minimally impactful to the model’s predictive power as compared to the influential connectivity and interaction-weighted geometry. We then evaluated the inverse condition by maintaining the protein geometry while ablating the network topology (SI Appendix, S7.1, available as supplementary data at *Bioinformatics* online). By randomizing edge placement while holding total node count, total edge count, average degree, and the exact edge-weight distribution invariant via Fisher–Yates permutation, we maintained overall network sparsity and bonding budget while destroying specific pairwise adjacencies, individual node degree profiles, and higher-order community structures. This manipulation led to a significant performance reduction across all metrics (Fig. 5), demonstrating that the model relies on evolved, non-random topological organization rather than global edge density alone. Because this topological noising strategy holds total edge count and global weight distribution strictly invariant, it acts as an implicit control for local contact density and overall edge frequency. Had the architecture relied merely on counting nearby contacts or aggregate bonding capacity, performance would have remained stable under edge rewiring. Instead, the sharp reduction in predictive accuracy confirms that StrucNS learns from the specific, non-random spatial organization of local contacts and higher-order topological architecture rather than simple contact counts. These results collectively demonstrate that network topology is a more potent predictor of protein stability than specific geometry. In this context, geometry serves primarily as a scaffold to establish the network topology or to recreate the latent physicochemical forces that drive protein folding and stability—a relationship that StrucNS successfully captures. Moreover, ablation of geometry in addition to ablating topology nominally but not substantially reduced performance relative to ablated topology alone (Fig. 5).

**Figure 5:**
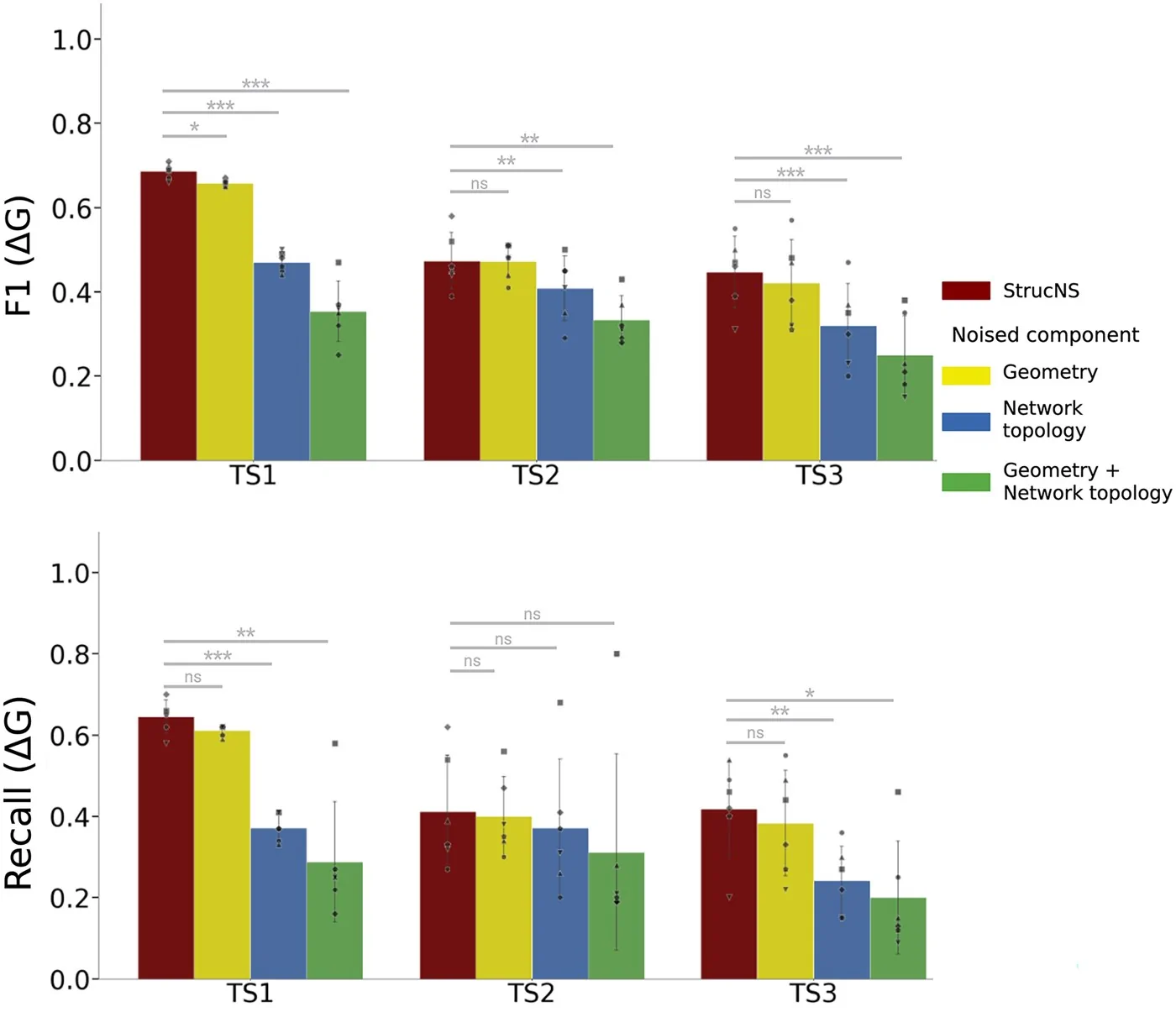
*Systematic ablation of geometry and network topology reveals that network topology is the primary locus of the stability signal.* The F1 (top) and Recall statistic (bottom) for predicting the stability classification (ΔG>3 kcal/mol) of variants are shown for StrucNS and comparative models with model elements ablated. Geometry indicates randomized three-dimensional node positions in the protein fold. Network topology indicates the connectivity map comprising nodes, edges, and edge weights without any spatial information regarding the nodes. Geometry was noised using a random walk approach (yellow). Network topology was noised by randomizing edges in the network while conserving network sparsity and ensuring all nodes remained connected (blue). Both geometry and network topology were randomized together (green) to ablate the effects of geometry and network topology simultaneously. In these two cases, geometry was randomized using random box (SI Appendix, S7.3, available as supplementary data at *Bioinformatics* online) and random walk (SI Appendix, S7.2, available as supplementary data at *Bioinformatics* online) approaches to ensure results were not an artifact of the randomization scheme. Finally, geometry was noised while peptide edges were maintained to reserve primary sequence information but exclude all non-peptide topology information.

#### 3.3.2 Mechanistic necessity of functional subnetwork assignment

To assess whether stability predictions depend on specific functional subnetwork definitions, we conducted a physicochemical label permutation experiment while maintaining the underlying graph topology and atomic spatial coordinates invariant. By uniformly shuffling the assignment of communities to random subnetworks, this ablation selectively decouples topological network position from residue chemical identity, isolating the predictive signal arising specifically from subnetwork-level physicochemical organization. Because direct residue-label randomization would disrupt structural integrity, this community-level ablation serves as a network-based equivalent that decouples chemical identity from 3D topology and geometry to highlight the contribution of physicochemically relevant subnetworks in the interaction-weighted network topology. This ablation of physicochemical assignments significantly reduced F1 and recall (Fig. 6). Most strikingly, performance on TS2, the de novo protein folds, was reduced to the level of noise (Fig. 2). Thus, physicochemical-based subnetwork assignments provide critical utility to the StrucNS approach.

**Figure 6:**
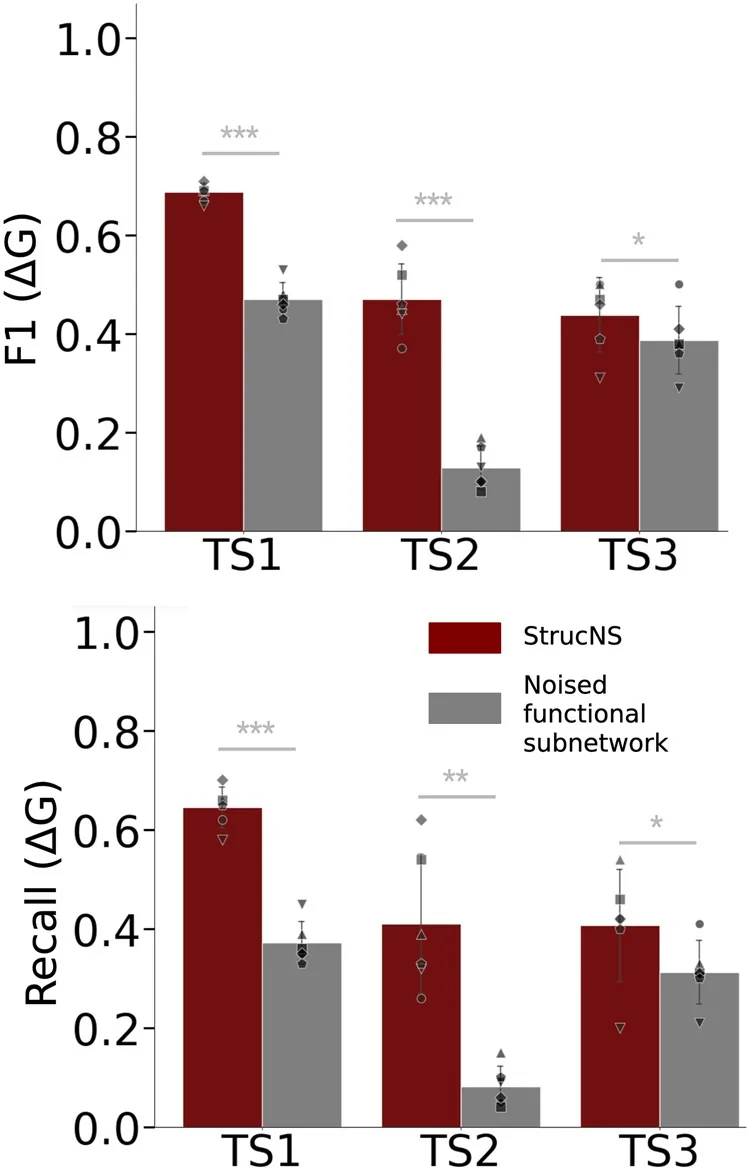
Ablation of functional subnetworks. The F1 statistic (top) and Recall (bottom) for predicting stability classification (ΔG>3 kcal/mol) are shown for StrucNS and a comparative model with specific elements ablated. Functional subnetworks are ablated by randomly assigning detected communities to subnetworks, rather than labeling them by function. Specifically, a community is randomly assigned to random subnetwork A, B, or C. It is then further assigned to either random subnetwork D or E, and finally, potentially randomized as part of random subnetwork F. These assignments are performed while keeping the total number of subnetworks conserved.

#### 3.3.3 Subnetwork-level vs. inter-subnetwork features

To resolve the multi-scale organization of the stability signal, we performed a hierarchical decomposition of the feature importance, categorizing SHAP contributions into subnetwork-level (aggregated community properties) and inter-subnetwork (pairwise interactions between functional modules) signals. Our results demonstrate that while the majority of the cumulative predictive power is stored in the aggregated properties of specific functional classes, the individual pairwise interactions exhibit a significantly higher average potency per feature (Fig. 4B). Analysis of the global importance share reveals that subnetwork-level features account for 75% of the total SHAP impact. Within this category, the model prioritizes the topological integrity of intermediate communities, as evidenced by the aforementioned dominance of degree and modularity in intermediate communities. Furthermore, the systematic appearance of percent polar across multiple subnetworks in the most influential features indicates that the model utilizes surface-core polarity distributions as a primary filter for identifying stably folded states. In contrast, inter-subnetwork (pairwise) features account for 25% of the importance share. This implies that the interaction between functional modules is also highly informative in the model. The prominence of ICFC metrics suggests that stability is sensitive to the specific “wiring” of the protein’s functional interface. These findings verify that the StrucNS framework operates through a dual-scale biophysical logic. The subnetwork-level features inform the macroscopic thermodynamic stability. Simultaneously, inter-subnetwork features capture the cooperative energy-coupling between distinct functional compartments.

#### 3.3.4 Impact of peptide and non-peptide interactions

To evaluate the specific contribution of primary sequence connectivity versus higher-order structural interactions, we conducted an ablation study focusing on the edge types within the RIN, illustrated in Fig. 7. By selectively removing either peptide (backbone) edges or non-peptide interactions, we quantify how the model’s prediction signal depends on interactions. Performance metrics across all test sets indicate that non-peptide interactions are the primary drivers of high-fidelity stability prediction. In TS1 and TS3, the removal of non-peptide edges results in a significant decline in the F1-score and recall. Conversely, the removal of peptide edges results in a negligible performance change in TS1, with F1-scores remaining stable at ∼0.68 as well as less significant drop in TS3 relative to non-peptide edge removal. This suggests that the model’s topological features are robust enough to infer the linear backbone path implicitly from the density of non-covalent clusters. Interestingly, in TS2, the performance of the model without peptide bond edges remains equivalent to the base model.

**Figure 7:**
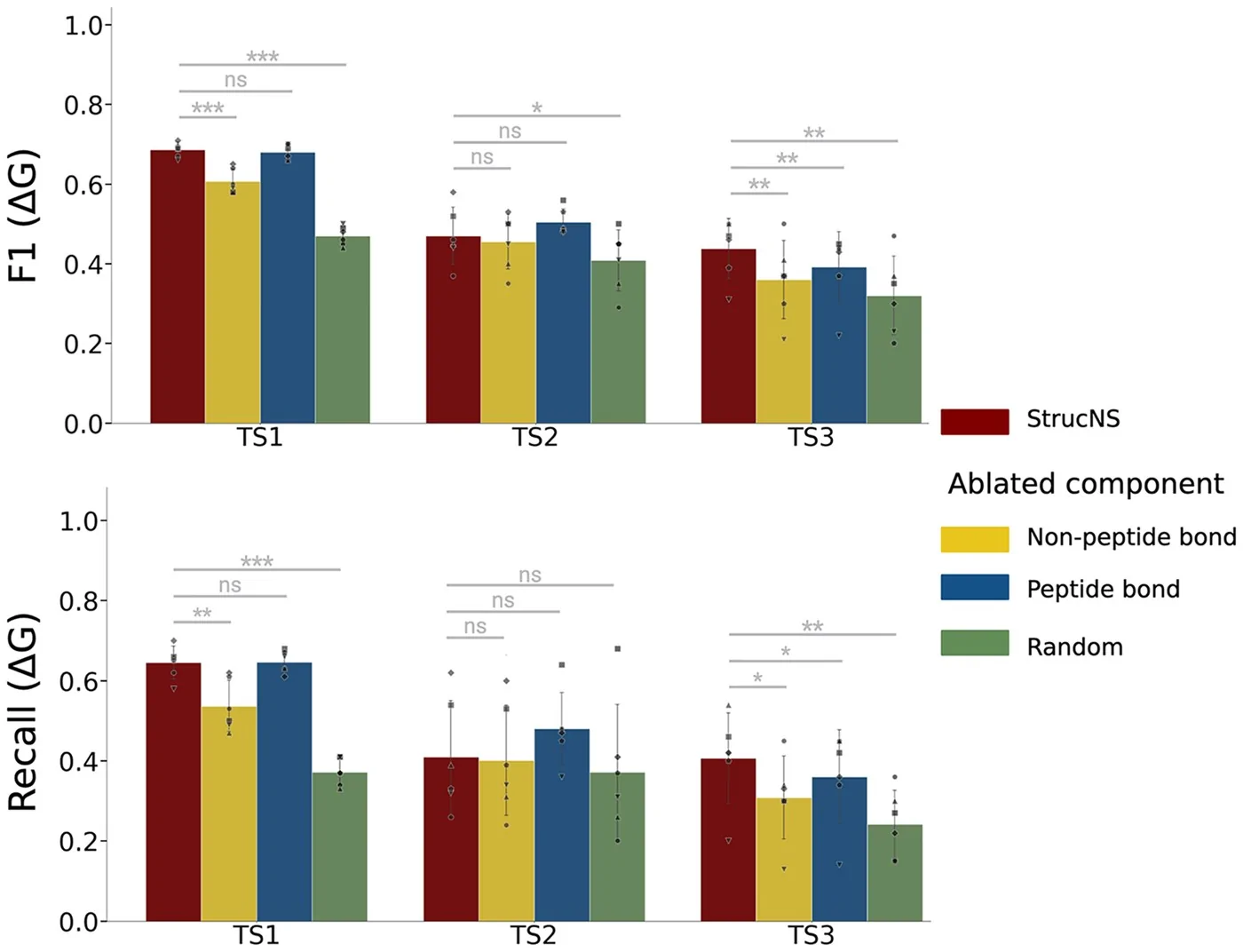
*Systematic ablation of interaction types.* The F1 (top) and Recall (bottom) for predicting stability classification (ΔG>3 kcal/mol) are shown for StrucNS and comparative models with specific elements ablated. The network topology comprises peptide edges and various non-peptide edges. Peptide edges represent the protein backbone chain, while non-peptide edges define the tertiary protein fold. Non-peptide edges were removed (yellow) to understand the importance of tertiary fold without backbone chain information. Conversely, peptide edges were removed (blue) to evaluate the backbone chain’s contribution. Finally, all interaction types were randomized (green) to provide a benchmark for noise.

The critical nature of non-covalent wiring is further highlighted in the distribution of metrics for TS2 and TS3. In TS2, removing these edges causes the recall to remain low (∼0.24–0.39), while in TS3, the ablated *non-peptide bond* regime results in a precipitous drop in recall for specific cases, significantly underperforming the base model. This indicates that for more challenging or “noisy” structural datasets, the model relies almost exclusively on non-covalent topological invariants to distinguish stable folds from decoys. Without these edges, the model lacks the necessary “cross-talk” features—such as ICFC and inter-modular edge density—required to maintain predictive equilibrium in the absence of a perfect crystal structure.

## 4 Discussion

In this study, we present StrucNS, a mathematical framework grounded in network theory that models protein fitness via interaction-weighted topology of the protein fold. While contemporary protein stability modeling is increasingly dominated by “black-box” architectures reliant on high-dimensional evolutionary embeddings, StrucNS offers a physically transparent alternative that derives its predictive signal directly from the explicit features of interaction-weighted network topology. By conceptualizing thermodynamic stability as an emergent property of network topology rather than a simple consequence of protein fold, we demonstrate that defined topological metrics serve as robust surrogates for the complex physicochemical forces governing the fitness landscape.

StrucNS leverages multiple previous innovations, including computational structure prediction (Wu *et al*. 2022), RINs (Amitai *et al*. 2004, Piovesan *et al*. 2016, Brysbaert and Lensink 2021), Louvain’s algorithm (Blondel *et al*. 2008), physicochemical labeling, graph feature perspectives, and a feedforward neural network, in a unique combination. StrucNS, efficiently trained on ∼500 000 datapoints, achieves competitive, and in many cases superior, performance compared to state-of-the-art PLMs within the context of the current test sets. It should be noted that StrucNS is trained on the ΔG > 3 kcal/mol test objective whereas the PLMs were not. Notably, in the *de novo* regime where evolutionary history is absent, StrucNS maintained a GMean of 0.55 while models like ESM-2 collapsed to 0.31 (Fig. 2), highlighting the resilience of a topology-based approach when navigating “fitness deserts.” Moreover, StrucNS was superior in predicting the likelihood of high stability in multi-mutants across a broad set of protein folds and sizes outside of the training regime (Fig. 3). Beyond mere prediction, the interpretability of StrucNS provides mechanistic insights into the structural logic of stability. SHAP analysis identifies the high connectivity and low modularity of intermediate (polar/hydrophobic mixed) subnetworks as primary predictors of stability. This suggests that the integration of the hydrophobic core with the polar surface through a highly connected intermediate region is more critical for structural integrity than the hydrophobic core alone.

Beyond elucidating fundamental insight of factors that dictate protein stability, StrucNS could empower protein stability engineering. Protein variants—identified via various search strategies (e.g. saturation mutagenesis, structure-guided mutagenesis, Bayesian optimization)—could be scored via StrucNS to identify mutants with increased likelihoods of high stability. In protein discovery workflows, StrucNS could be employed to evaluate the stability of a designed library or aid in scoring subsets of sequence space before proceeding to experimental validation. The mechanistic insight of StrucNS could also be leveraged to guide stabilization. StrucNS could be applied to identify the specific underperforming topological features that drove the designs toward instability. This feedback loop enables researchers to rationally identify residues for diversification within specific subnetworks, effectively shortening the design cycle. Future work can evaluate the efficacy of such techniques.

StrucNS benefits from the deep, high-integrity Megascale dataset of Tsuboyama et al. (Tsuboyama *et al*. 2023), which includes a broad set of protein folds and sequences, albeit focused on relatively small proteins (40–72 amino acids). Yet, the current implementation of StrucNS outperforms more broadly trained protein language models including on multi-mutants of a broad size range (Fig. 3). As additional datasets emerge, updated training can be performed to evaluate mechanistic insights—as well as predictive performance—across an even more expansive set of proteins. More broadly, the StrucNS network topology approach can be applied to other modes of protein performance including binding or additional metrics of developability. Several other limitations of the current framework warrant note. StrucNS requires an explicit three-dimensional structure, whereas sequence-based PLMs operate on primary sequence alone. Our geometric noising controls indicate robustness to random coordinate perturbation (Fig. 5), but a systematic structure-prediction bias affecting a particular fold class would not be detected by those controls. The feature space is hand-constructed. The 294 features, the physicochemical labeling thresholds, and the weights assigned to each interaction type encode design decisions informed by biophysical reasoning rather than learned from data. PLMs require no such curation, and it constrains the framework’s capacity to capture determinants not anticipated during feature design. Third, training was restricted to miniproteins of 40–72 residues; although StrucNS generalizes to substantially larger globular proteins (Fig. 3), the training distribution remains narrow relative to the structural universe. Finally, we employed classification rather than regression to mitigate overfitting on high-throughput data with experimental variability; regression on continuous ΔG remains a natural extension as datasets grow.

The current performance and insight from StrucNS emerged from careful design of model architecture and components. Despite the advancements, several avenues for future refinement remain. We utilized a classification approach, outputting the likelihood of stability being greater than 3 kcal/mol, to mitigate potential overfitting on high-throughput datasets with experimental variability. A stricter threshold was maintained to ensure a robust stable class. Future iterations may explore regression models as larger datasets become available. While feature analysis and ablation analysis revealed the relative impact of numerous design choices, further optimization can also be performed. Low-impact features, identified via SHAP analysis (Fig. 4), could be removed to enable more efficient training of impactful features. Interaction identification (distance thresholds and residue identities) and strengths can be tuned, and additional interaction types could be evaluated. Additional community classifications could also be tested. It is acknowledged that the thoughtfully-selected features of StrucNS are a noted inefficiency relative to the simple inputs of PLMs. Yet, an opportunity exists: by integrating topological perspectives with emerging PLMs, one could develop synergistic models that combine the breadth of evolutionary grammar with the precision of structural network science toward a map of the protein fitness landscape.

## Supplementary Material

btag718_Supplementary_Data
