# Supplementary material for: StrucNS reveals interaction-weighted network topology as the driving predictor of absolute stability of natural and de novo proteins
Source: Bioinformatics. 2026 Sep 30;42(10):btag718. doi: 10.1093/bioinformatics/btag718 (PMC13679978; doi:10.1093/bioinformatics/btag718)
Supplement: Supplementary file 1 [file btag718_supplementary_data.pdf]

Supplementary Information

S1. StrucNS details

S1.1. Network Construction and Interaction Criteria

The Residue Interaction Network (RIN) was generated using a custom Python pipeline incorporating the Biopython PDB parser and NetworkX. For all protein variants in our mutational libraries, 3D structural coordinates were generated via OmegaFold Wu et al. (2022), as these high-fidelity predictions provide a consistent geometric basis for evaluating mutations in the absence of experimental structures. Residues were represented as nodes located at the centroid of all non-hydrogen atoms.

Edges were classified into six types based on distance cutoffs and atomic contact criteria. To ensure biophysical accuracy, aromatic interactions were identified using a centroid-to-centroid distance cutoff of 4.5 Å between aromatic rings (PHE, TRP, HIS, TYR), serving as a geometric proxy for orientations that enable π-π stacking. Further specific criteria for each interaction type are detailed below:

- **Peptide Bonds:** Defined between residues i and $i+1$ along the polypeptide chain.
- **Hydrophobic Interactions:** Established between side-chain atoms of non-polar residues using a centroid-to-centroid distance cutoff of 5.0 Å.
- **Hydrogen Bonds:** Identified between donor and acceptor atoms with an atom-to-atom distance < 3.5 Å (4.0 Å for sulfur-containing donors).
- **Disulfide Bridges:** Defined between SG atoms of CYS residues within a 2.2 Å threshold.
- **van der Waals Forces:** Modeled as non-specific steric contacts for all residue pairs with a centroid distance < 5.0 Å not meeting the above specific criteria.

S1.2. Weighting Schema and Energetic Justification

To model the relative rigidity and thermodynamic contribution of the protein fold, weights (w) were assigned based on representative bond dissociation energies (ΔH_f) and empirical stability scales. The high weight assigned to peptide bonds ($w = 10$) enforces the primary backbone constraint as the most rigid component of the network. Non-covalent interactions were weighted to reflect their relative contributions to fold stability: hydrophobic contacts ($w = 0.55$) represent entropic core stabilization; hydrogen bonds ($w = 0.28$) represent polar stabilization; disulfide bridges ($w = 1.0$) account for covalent cross-links; aromatic stacking ($w = 0.2$) and van der Waals forces ($w = 0.1$) represent weaker, orientation-dependent or non-specific steric effects.

S1.3. Community Detection (CD) via Modularity Maximization

The structural network was partitioned into communities using the Louvain heuristic, a two-phase iterative algorithm designed to maximize the modularity index (Q). In this framework, modularity serves as an objective function that quantifies the quality of a particular division of the network into communities.

For a weighted graph, Q is defined as the difference between the actual weight of edges within communities and the expected weight if edges were placed at random while preserving the degree sequence of the nodes (the "background connectivity" null model). Mathematically, for a weighted network, modularity is expressed as:

$$Q = \frac{1}{2m} \sum_{i,j} \left[A_{ij} - \frac{k_i k_j}{2m} \right] \delta(c_i, c_j) \tag{S1}$$

Where:

- A_{ij} is the weight of the edge between residue nodes i and j.
- $k_i = \sum_j A_{ij}$ is the sum of the weights of the edges attached to node i (weighted degree).
- $m = \frac{1}{2} \sum_{i,j} A_{ij}$ is the total sum of all edge weights in the network.
- c_i and c_j represent the community assignments of nodes i and j, respectively.

- $\delta(c_i, c_j)$ is the Kronecker delta function, which equals 1 if $c_i = c_j$ and 0 otherwise.

The algorithm iteratively optimizes Q through two repeating phases: (i) local moving of nodes between communities to maximize local modularity gain, and (ii) aggregation of nodes assigned to the same community into a single "super-node" to form a new network for the subsequent pass. This variable assignment process continues until no further increase in Q is possible. This naturally discovers the optimal number of communities (K) for a given protein of N residues, where the condition $K < N$ facilitates the transition from individual atomic centers to higher-order structural modules without the premature loss of structural information.

S1.4. Hierarchical Spring-Balance Layout

To preserve the relative spatial orientation of structural modules while emphasizing interaction density, we implemented a hierarchical spring-balance layout using a modified Fruchterman-Reingold algorithm. The process is divided into two discrete tiers of optimization to reach a global topological equilibrium:

1. **Tier 1: Global Community Positioning.** A "community graph" is constructed where each node represents a coarse-grained community (K). Edge weights between community nodes i and j are calculated as the sum of all inter-community bond weights: $W_{ij} = \sum_{u \in C_i, v \in C_j} A_{uv}$. The global positions of these modules are determined by a 3D spring layout. By using the original PDB centroids as the initial state for the simulation, we maintain the native global topology while allowing the modules to shift toward a state of balanced tension.
2. **Tier 2: Local Residue Relaxation.** For each community C_k, the internal residues are positioned relative to one another using a local spring-layout simulation. The internal edge weights A_{uv} (where $u, v \in C_k$) define the attractive spring constants. Once local equilibrium is reached, the residues are translated such that their local centroid aligns with the global community position calculated in Tier 1.

The force F acting on a node in this system is defined by the summation of attractive forces f_a (along edges) and repulsive forces f_r (between all node pairs):

$$f_a(d) = \frac{w_{uv} \cdot d^2}{k}, \quad f_r(d) = \frac{k^2}{d} \tag{S2}$$

where d is the distance between nodes, w_{uv} is the physicochemical bond weight, and k is the optimal distance parameter derived from the expected node density. This hierarchical approach ensures that the resulting "topological coordinates" reflect the physical equilibrium of the interaction network. By allowing nodes to migrate under these forces, the layout acts as a noise-reduction filter; residues that appear buried in a noisy structure but lack strong stabilizing connectivity will drift toward the periphery, while those forming the true structural core will be pulled closer together.

S1.5. Multi-Label Functional Classification Logic

To translate topological communities into biologically interpretable modules, we categorized each community C based on the intrinsic properties of its constituent residues. This classification relies on two primary biophysical scales: the Kyte-Doolittle scale, which measures desolvation energy, and the Grantham scale, which accounts for side-chain polarity and volume.

For each community, a Hydrophobicity Score (H_C) was calculated as the arithmetic mean of the normalized scores of its residues. To ensure the classification was robust against structural noise, we applied a three-tiered labeling logic:

1. **Hydrophobicity Regime:** Each community is assigned exactly one label based on its mean score H_C:
   - **Hydrophobic:** $H_C \geq 0.66$, identifying dominantly non-polar core fragments.
   - **Polar:** $H_C \leq 0.33$, identifying dominantly hydrophilic surface fragments.
   - **Mixed:** $0.33 < H_C < 0.66$, identifying amphipathic or transition regions.
2. **Charge Status:** Each community is assigned exactly one label based on the fraction of ionizable residues (f_{charge}):

- **Charged:** If $f_{charge} > 0.3$ (where residues are Arg, Lys, Asp, or Glu).
- **Uncharged:** If $f_{charge} \leq 0.3$.

3. **Flexibility Propensity:** A community is tagged as **Flexible** if the fraction of residues associated with high conformational entropy (f_{flex}, specifically Gly and Pro) exceeds 30% ($f_{flex} > 0.3$).

This framework ensures that every module is described across three orthogonal biophysical axes. For example, a module might be classified as "Hydrophobic-Uncharged" while another is "Polar-Charged-Flexible." This nuanced characterization is necessary to preserve the subtle biophysical nuances of the protein fold during the subsequent feature aggregation steps, where topological invariants are mapped to these functional categories.

S1.6. Hierarchical Aggregation Logic

The transformation from raw residue data to the final machine-learning-ready feature vector follows a two-step hierarchical protocol.

S1.6.1. Step 1: Node-to-Community Aggregation

For a community C containing a set of nodes V_C, the community-level feature f_C is calculated as:

$$f_C = \frac{1}{|V_C|} \sum_{i \in V_C} x_i \tag{S3}$$

S1.6.2. Step 2: Community-to-Class Aggregation

Communities are grouped by physicochemical labels. We derive class-level features F_{Class} via three strategies:

- **Node-Weight Mapping (NWM):** $F_{NWM} = \frac{\sum (f_C \cdot |V_C|)}{\sum |V_C|}$
- **Volume-Weight Mapping (VWM):** $F_{VWM} = \frac{\sum (f_C \cdot Vol_C)}{\sum Vol_C}$
- **Simple Mean (SM):** $F_{SM} = \frac{1}{K} \sum_{j=1}^{K} f_{C_j}$

S1.7. Comprehensive Feature Definitions

To ensure reproducibility and clarity, this section provides the formal mathematical definitions of the features extracted from the residue interaction networks. These features are organized into four functional categories and aggregated using three distinct strategies: **Node-Weighted Mean (NWM)**, **Volume-Weighted Mean (VWM)**, and **Simple Mean (SM)**.

S1.7.1. Aggregation Strategies

The community-level metrics (F_k) are aggregated into class-level descriptors (F_C) as follows:

- **Node-Weighted Mean (NWM)**: $F_{NWM} = \frac{\sum_{k \in C} F_k \cdot n_k}{\sum_{k \in C} n_k}$, where n_k is the number of residues in community k.
- **Volume-Weighted Mean (VWM)**: $F_{VWM} = \frac{\sum_{k \in C} F_k \cdot V_k}{\sum_{k \in C} V_k}$, where V_k is the spring-model convex hull volume.
- **Simple Mean (SM)**: $F_{SM} = \frac{1}{|C|} \sum_{k \in C} F_k$, representing the unweighted average across all communities in class C.

—

S1.7.2. 1. Connectivity Based Features

These metrics quantify the topological robustness and communication efficiency of the structural subnetworks.

- **Weighted Degree (D)**: The average sum of edge weights w_{ij} incident to residues within the community.
- **Clustering Coefficient (CC)**: Measures the local transitivity within the community graph.
- **Centrality Metrics**: Includes *Betweenness Centrality* (shortest-path control), *Eigenvector Centrality* (influence), and *PageRank* (importance based on neighbor quality).

- **K-Core Decomposition**: The level of the maximal subgraph where all residues have at least k neighbors.
- **Modularity (M)**: The degree to which the community structure is optimized relative to a null model.
- **Edge/Bond Density**: *Edge Density* is the ratio of existing to potential edges; *Bond Density* is the average weight per edge: $BD = \frac{\sum w_{ij}}{|E|}$.
- **Algebraic Connectivity (λ_2)**: The second smallest eigenvalue of the Laplacian matrix, indicating subnetwork synchronization and stability.
- **Topological Density Factor (TDF)**: A hybrid metric defined as the ratio of topological edge density to the physical convex hull volume: $TDF = \frac{\rho_{edge}}{V_{hull}}$.
- **Inter-Class Boundary Fraction (ICBF)**: Ratio of the sum of weights for edges connecting Class A and B to the total weighted degree (perimeter) of the boundary nodes.
- **Class Territorial Overlap (CTO)**: The volumetric intersection ratio between two class territories: $CTO = \frac{\min(V_a, V_b)}{\max(V_a, V_b)}$.
- **Inter-Class Force Coefficient (ICFC)**: The total interaction weight between classes normalized by the maximum individual bond weight observed.

S1.7.3. 2. Geometry Based Features

Metrics focused on the physical distances and spatial resolution of the residue clusters.

- **Average Pairwise Distance (APD)**: The mean Euclidean distance between all possible pairs of residues in the community.
- **Network Resolution Factor (NRF)**: The ratio of the spatial spread in PDB coordinates (σ_{PDB}) to the spread in spring-relaxed coordinates (σ_{spring}): $NRF = \frac{\sigma_{PDB}}{\sigma_{spring}}$.
- **Curvature Correction Ratio (CCR)**: The ratio of compactness between the native and equilibrated states: $CCR = \frac{1/\sigma_{PDB}}{1/\sigma_{spring}}$.
- **Centroid-Induced Coordinate Shift (CICS)**: The Euclidean distance between the community's PDB-centroid and its spring-model centroid.
- **Class Topological Stability (CTS)**: The ratio of inter-community weights to intra-community weights within a functional class.

S1.7.4. 3. Projection Based Features

These features project structural data into 3D spatial descriptors to characterize the global protein envelope.

- **Convex Hull Volume (V_{ch})**: The volume of the smallest convex polyhedron containing all residue coordinates in the community.
- **Spread (σ)**: The standard deviation of residue distances from the community centroid.
- **Proximity Centralization**: A measure of subnetwork density relative to its geometric center: $PC = \frac{\sum 1/dist(i, centroid)}{n-1}$.
- **Structural Tightness Coefficient (STC)**: The fractional volume occupied by a class relative to the total volume of the complex.
- **Z-Centroid (Z_{cent})**: The distance from the community centroid to the global center of mass of the protein.
- **Centroid Coordinates (X, Y, Z)**: The mean spatial projection of the class in 3D space.
- **Weighted Inter-Class Distance Spread (WICDS)**: The standard deviation of all pairwise Euclidean distances between residues of two different functional classes.

S1.7.5. 4. Chemical and Functional Features

- **Polar Percentage ($PolarPct$)**: The fraction of residues in the community belonging to polar or charged categories (GLU, ASP, LYS, ARG, HIS).

S2. Additional results

S2.1. Distribution of Functional Subnetworks vs. SASA

To characterize the structural environment of the predicted functional subnetworks, we quantified the solvent accessibility of each constituent residue across the three physicochemical classes. Solvent Accessible

Surface Area (SASA) was calculated using the Shrake-Rupley algorithm as implemented in the Biopython structural bioinformatics package. To account for the inherent size differences among amino acid side chains, raw SASA values were converted to Relative Solvent Accessibility (RSA) by normalizing against the maximum possible SASA (S_{max}) for each residue type in a Gly-X-Gly tripeptide context, using reference values from Miller et al. (1987). This normalization yields a scale where 0.0 represents a fully buried residue and 1.0 represents maximum solvent exposure.

The resulting structural consensus, derived from $n = 17,135$ protein environments, reveals distinct spatial partitioning (Fig. S1). The *Hydrophobic* subnetwork exhibits a sharp density peak at low RSA values near 0.2, confirming that these predicted functional nodes predominantly localize within the protein core to drive stability via the hydrophobic effect. Conversely, the *Polar* subnetwork displays a significant rightward shift with a density maximum at approximately 0.6 RSA, consistent with the requirement for polar residues to reside on the protein surface to maintain solubility and facilitate hydrogen-bonding with the aqueous solvent. The *Mixed* subnetwork serves as a structural intermediate, with a broad distribution centered near 0.55, suggesting that these residues form a transitional interface between the hydrophobic interior and the solvent-exposed exterior. The relationship between the localized residue class and its solvent environment is captured by the mean Kernel Density Estimate (KDE) for each class c, calculated as:

$$\bar{f}_c(x) = \frac{1}{N} \sum_{i=1}^{N} \mathsf{KDE}_i(x) \tag{S4}$$

where x represents the normalized RSA and N is the number of proteins. The observed robustness of these distributions demonstrates that the deep learning model's predicted features are grounded in fundamental principles of protein architecture. These results provide biophysical validation that the functional subnetworks identified by the model correspond to specific, structurally conserved niches within the protein fold.

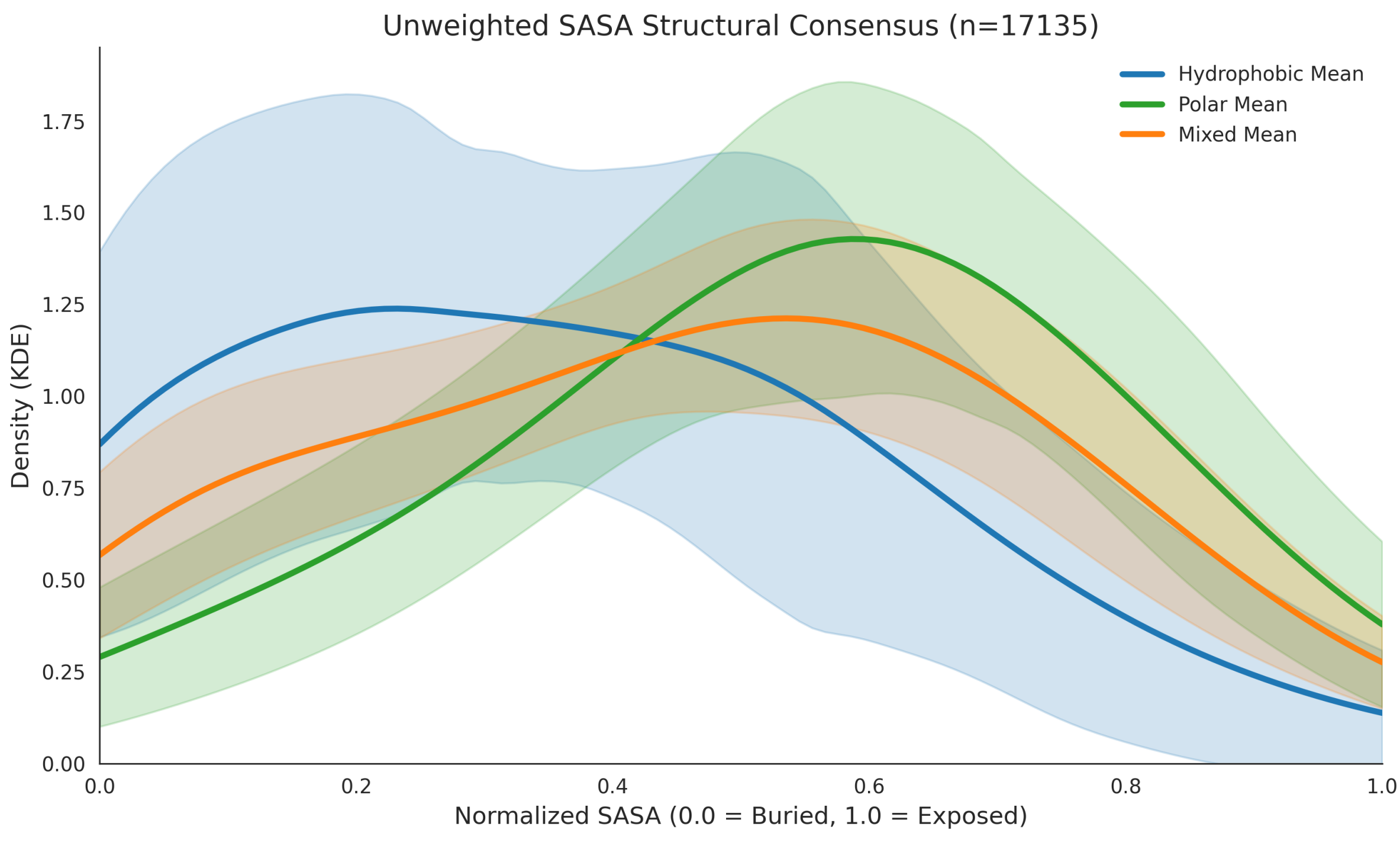

Figure S1 Structural consensus of SASA distributions for predicted functional subnetworks. The plot illustrates the unweighted mean KDE densities (solid lines) and standard deviations (shaded regions) for Hydrophobic (blue), Polar (green), and Mixed (orange) residue classes ($n = 17,135$). The clear spatial segregation between the buried hydrophobic core and the exposed polar surface validates the model's ability to discern structurally relevant residue environments.

S2.2. Monitoring overfitting

To evaluate the stability and generalizability of the deep learning architecture, we monitored the training dynamics across the cross-validation folds. Figure S2 illustrates the evolution of the primary performance metrics for the optimal hyperparameter configuration for one of the six ensemble models of StrucNS. All of the six model ensembles followed the same pattern while monitoring for overfitting. The *Average Training Loss* (solid blue line) demonstrates a monotonic decay, asymptotically approaching a minimum value near 0.225, which signifies effective minimization of the objective function.

Parallel to the training trajectory, the *Average Validation Loss* (orange dashed line) exhibits a rapid initial descent before stabilizing at approximately epoch 20. The absence of a characteristic "U-shaped" divergence in the validation loss confirms that the model successfully mitigated overfitting, maintaining a minimal generalization gap. This stability is largely attributable to the implementation of family-aware data partitioning and the optimized dropout-regularization layers.

Classification performance was primarily assessed via the *Average* F_1 *Score* (solid green line), which provides a balanced metric for identifying high-stability variants ($\Delta G > 3$). As shown in the dual-axis plot, the F_1 score experiences a sharp increase during the first 15 epochs, eventually plateauing at a robust value of approximately 0.73. The synchronization between the stabilization of the validation loss and the plateauing of the F_1 score indicates a well-calibrated learning rate and effective feature extraction.

The optimization objective, defined as the *Mean Maximal* F_1 ($\bar{F}_{1max}$), was calculated to ensure the final model captured the peak discriminatory power achieved during the training process:

$$\bar{F}_{1max} = \frac{1}{K} \sum_{k=1}^{K} \left(\max_{e \in \{1,\ldots,E\}} F_1(k,e) \right) \tag{S5}$$

where $K = 5$ represents the cross-validation folds and E is the epoch count. To preserve the most generalizable parameter state, we utilized a weight-restoration protocol that isolated the specific epoch e^* corresponding to the global maximum of the validation F_1 curve. This methodology ensures that the final predictive model represents the optimal compromise between sensitivity and specificity before the onset of marginal stochastic fluctuations observed in the later epochs ($e > 35$).

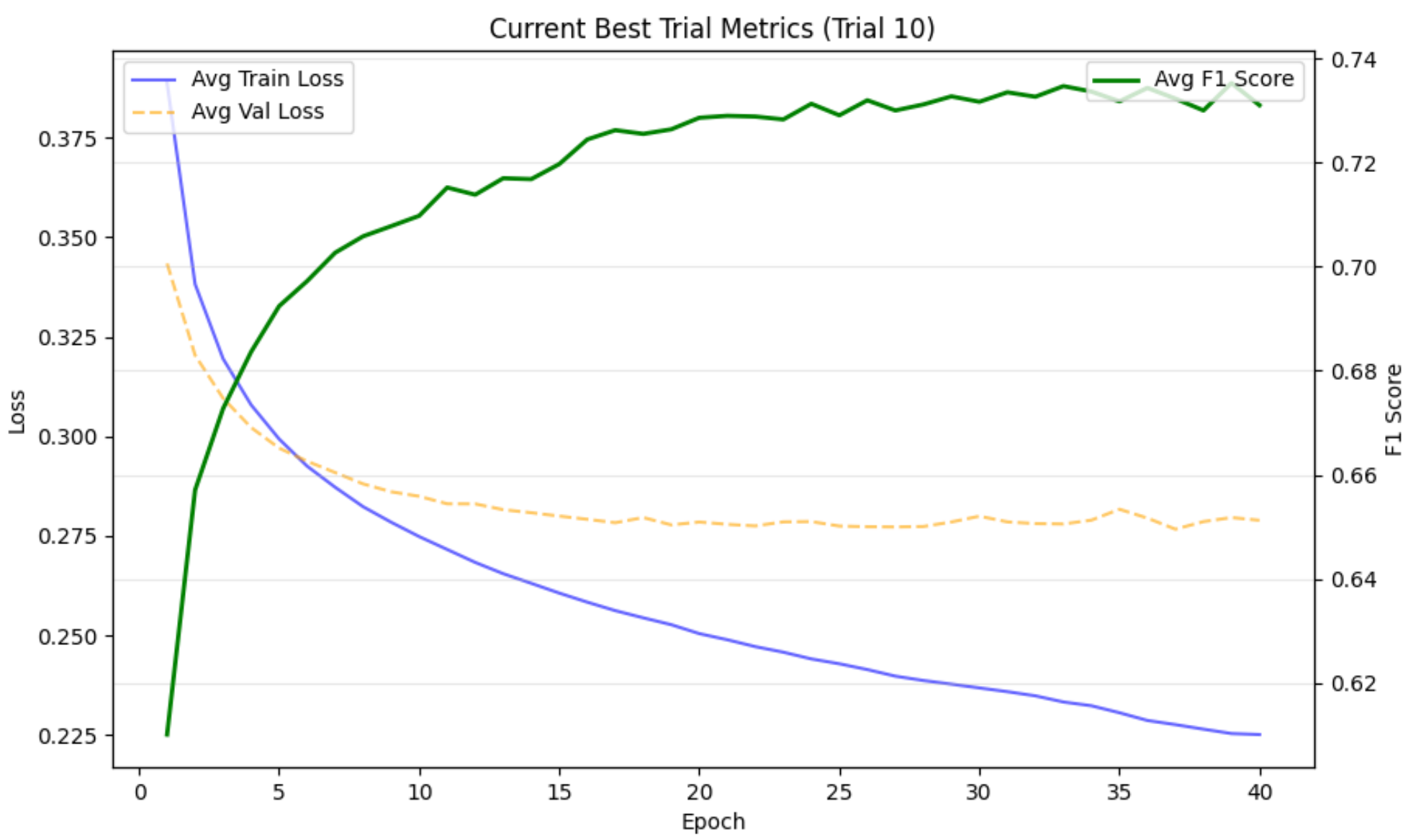

Figure S2 Training and validation metrics for the optimized ΔG prediction model. The primary y-axis (left) displays the binary cross-entropy loss for training (solid blue) and validation (dashed orange) sets. The secondary y-axis (right) denotes the F_1 score (solid green) achieved across the validation folds. Convergence is reached near epoch 40, where the F_1 score stabilizes at ≈ 0.73, indicating high model reliability in identifying stable variants.

S3. Sensitivity analysis of classification thresholds

S3.1. ΔG threshold

We selected $\Delta G > 3$ kcal/mol as the definition of stable. The Megascale dataset, as with all experimental measurements, carries measurement uncertainty (Figure S3): the 95% confidence interval on ΔG has a median width of only 0.17 kcal/mol but a long right tail, reaching 2.21 kcal/mol at the 99th percentile (We filtered the dataset to have CI breadth < 3 kcal/mol). A nominal $\Delta G > 0$ kcal/mol threshold, the definition of thermodynamic stability, sits well inside this noise band for a non-trivial fraction of variants, meaning many "stable" calls near zero would plausibly be measurement noise rather than genuine stability. $\Delta G > 3$ kcal/mol was chosen as a conservative buffer comfortably above the 99th-percentile CI width, so that positive-class labels correspond to variants whose stability is unlikely to be an artifact of experimental noise and correspond to the magnitude of stabilization that is functionally meaningful for downstream engineering applications rather than marginal thermodynamic stability.

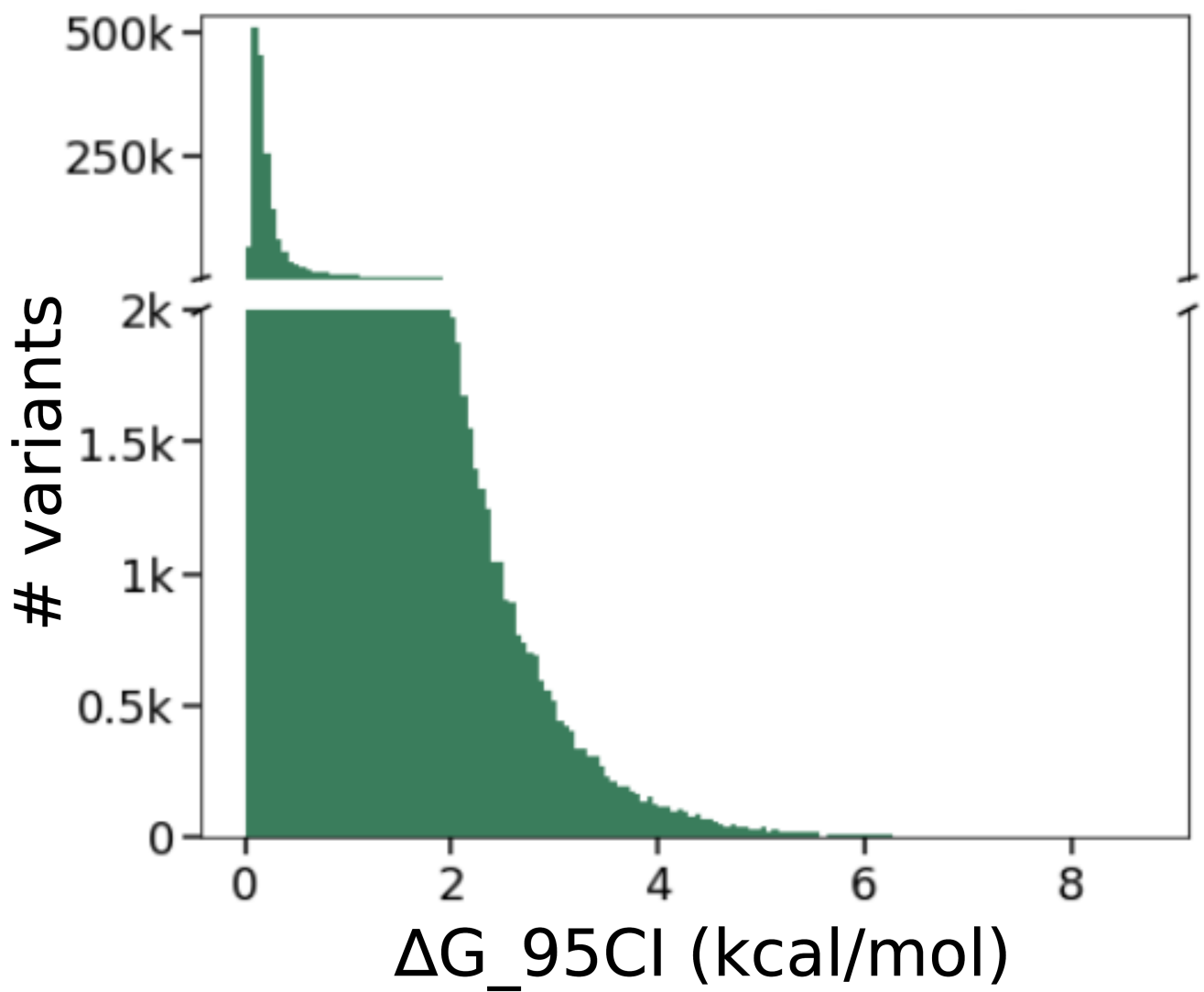

Figure S3 Histogram of the width of the 95% confidence interval for variants in the Megascale dataset.

This choice has a direct, quantifiable consequence for the composition of each test set: as the threshold tightens from $\Delta G > 0$ to $\Delta G > 4$ kcal/mol, the fraction of variants labeled "stable" falls steadily from 79–83% (0 kcal/mol) to 39–51% (2 kcal/mol) to 23–29% (our chosen 3 kcal/mol) to 11–12% (4 kcal/mol), consistently across TS1, TS2, and TS3 (Figure S4). We report this explicitly because it has direct bearing on the interpretation of raw classification metrics. Both precision-based statistics and F1 are well known to be inflated by a dominant positive class independent of genuine discriminative performance Chicco and Jurman (2020); this is exactly what we observe in our own data. At $\Delta G > 0$, where the positive class comprises 79–83% of each test set, StrucNS achieves F1 of 0.89–0.91 while its Recall on the negative (unstable) class collapses to just 0.25–0.38 — meaning the model correctly identifies only a quarter to a third of genuinely unstable variants, yet this failure is almost entirely masked by the high F1 score because negatives are rare (Figure S5).

For this reason, we regard the geometric mean of sensitivity and specificity (GMean) as the more trustworthy metric for threshold selection, following its original introduction for imbalanced classification evaluation Kubat et al. (1997) and its subsequent endorsement in the broader imbalanced-learning literature as a metric that, unlike accuracy or F1, penalizes a classifier equally for failing on either class regardless of class prevalence He and Garcia (2009). By this metric, Gmean is not maximized at our chosen threshold: it peaks instead at $\Delta G > 2$ kcal/mol for both models on all three test sets (StrucNS: 0.77–0.81 at 2 kcal/mol vs. 0.59–0.77 at 3 kcal/mol; ESM-2: 0.71–0.94 at 2 kcal/mol, its highest value in every one of the six threshold-by-test-set comparisons) (Figure S4). We attribute this to a near-balanced positive/negative split (39–51% positive), which is the regime in which balanced-accuracy-style metrics are easiest to optimize; both more permissive and more stringent thresholds move away from balance in opposite directions and both degrade Gmean accordingly.

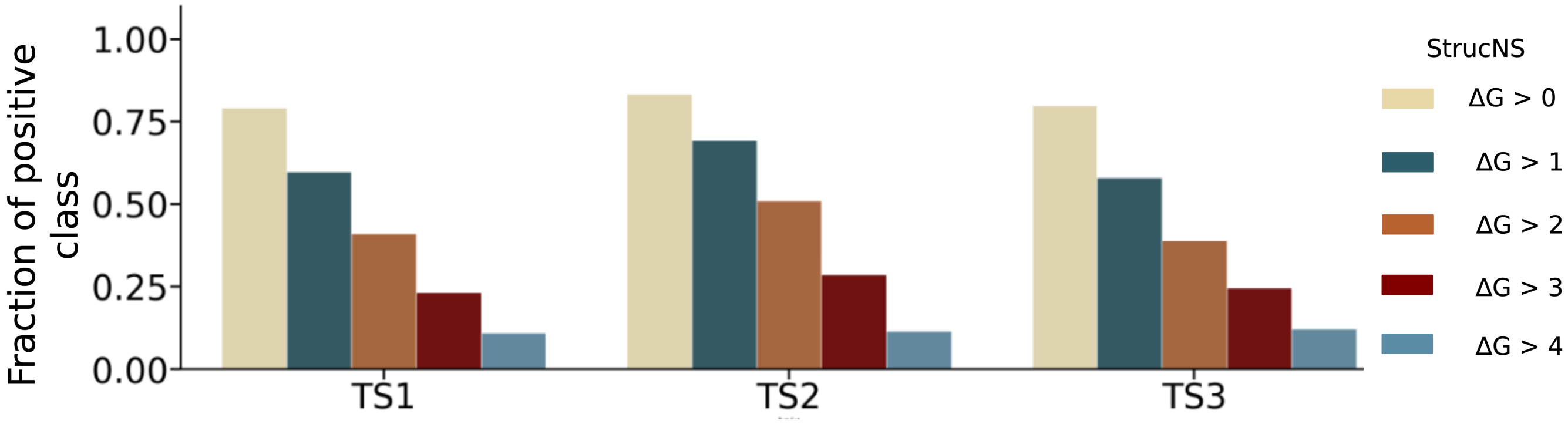

Figure S4 The fraction of variants within the positive class (Δ G $>$ threshold) is presented for each test set.

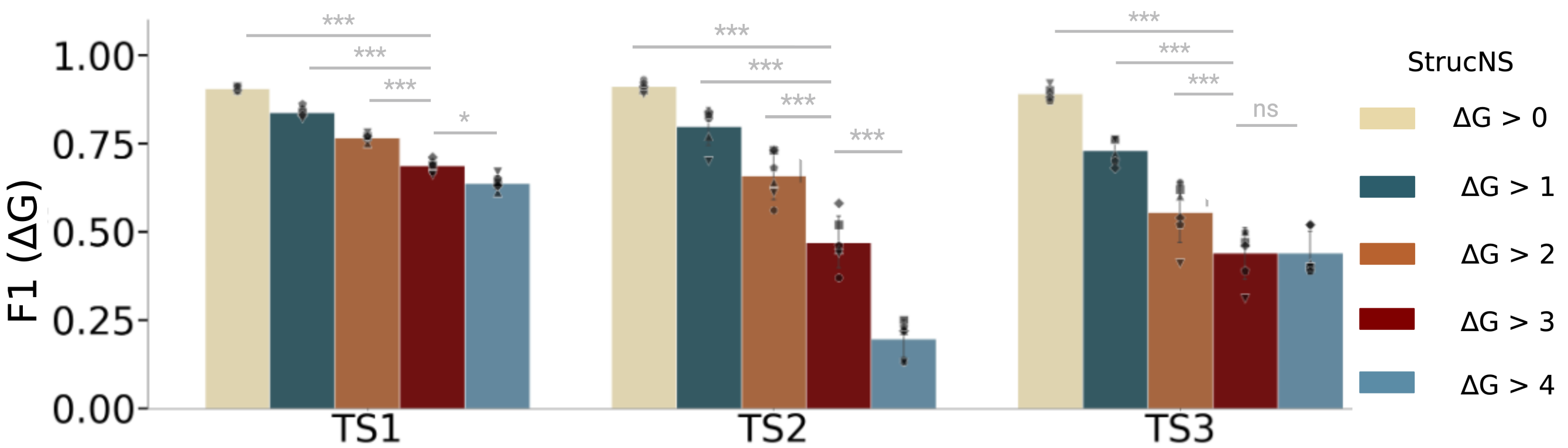

Figure S5 Model performance is impacted by the stability threshold. StrucNS was trained and tested with the indicated ΔG threshold. The F1 statistic for predicting the stability classification for each test set is reported as the mean $\pm$standard deviation across six train-test splits.

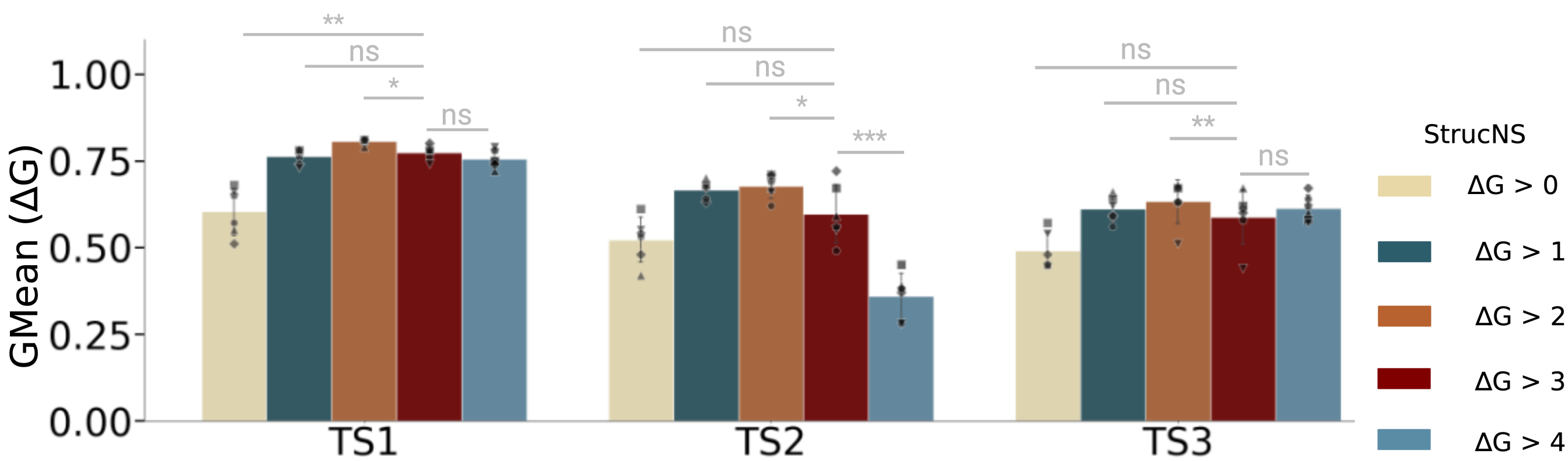

Figure S6 Model performance is impacted by the stability threshold. StrucNS was trained and tested with the indicated ΔG threshold. The Gmean for predicting the stability classification for each test set is reported as the mean $\pm$standard deviation across six train-test splits.

We highlight two implications of this analysis. First, because the Gmean-maximizing threshold (2 kcal/mol) and the F1-maximizing threshold (0 kcal/mol) both differ from our chosen $\Delta G > 3$ kcal/mol, we note that the conservative threshold was not selected to flatter either model's reported performance — by either metric, a different, more favorable threshold was available and not used. Second, because the same qualitative pattern (Gmean peaking near a balanced split, F1 tracking prevalence, negative-class recall collapsing under loose thresholds) replicates independently in the supervised ESM-2 baseline evaluated on identical data splits, we conclude this is a property of the labeling scheme itself rather than an artifact specific to our architecture. Consequently, comparisons between StrucNS and ESM-2 at $\Delta G > 3$ kcal/mol are not differentially biased toward either model — both are evaluated under the same prevalence conditions

and both show the same direction and approximate magnitude of metric sensitivity to threshold choice. We selected $\Delta G > 3$ kcal/mol because it reflects the rarity of robustly, functionally stabilizing mutations expected in real protein engineering settings, and because it sits safely above the noise of the underlying experimental measurements, not because it was the threshold most favorable to either model's reported metrics.

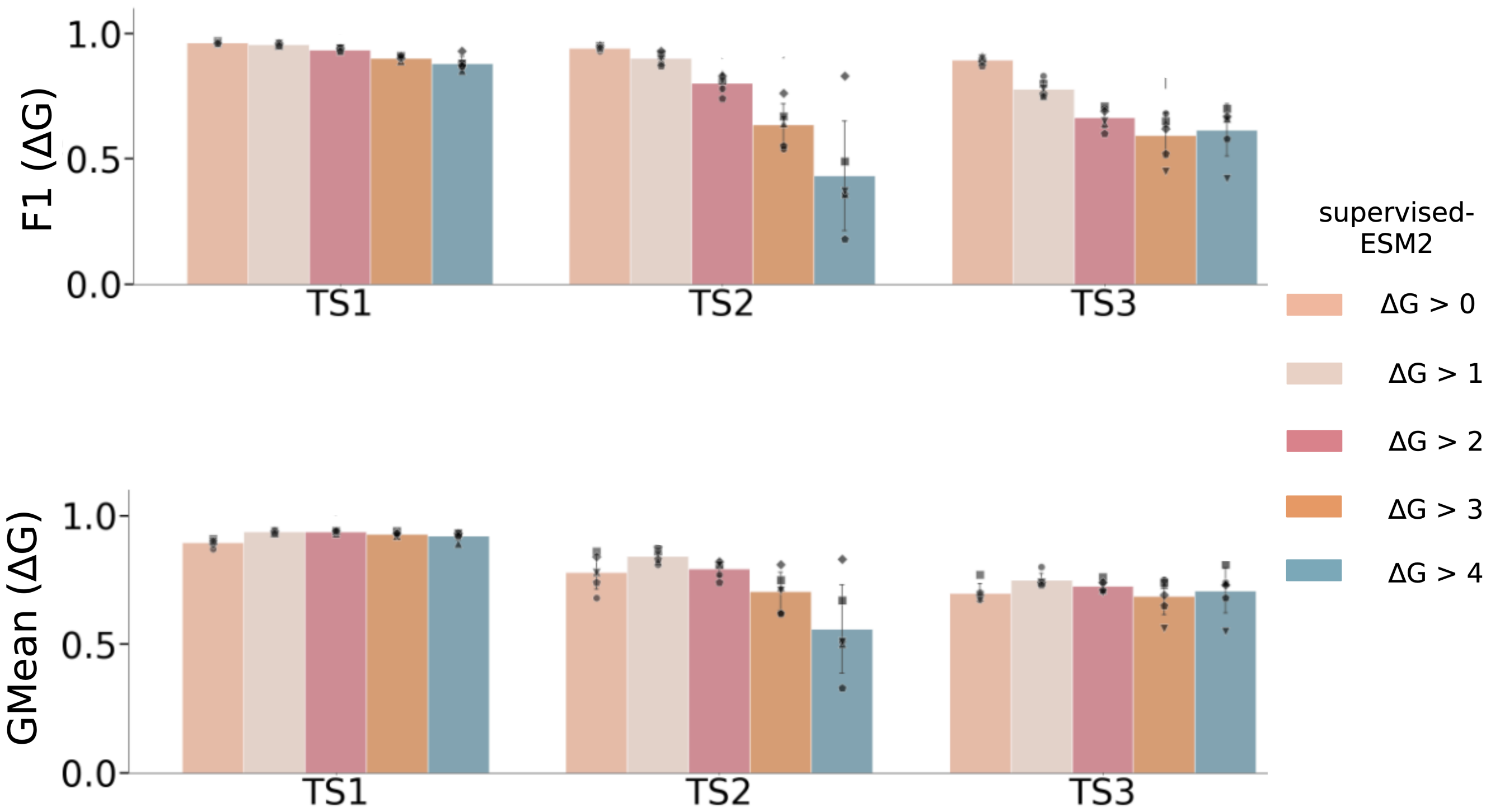

Figure S7 Model performance is impacted by the stability threshold. Supervised-ESM2 was trained and tested with the indicated ΔG threshold. The F1 and GMean statistics for predicting the stability classification for each test set is reported as the mean $\pm$standard deviation across six train-test splits.

S3.2. LLR threshold

Across the three test sets, perturbing the LLR decision threshold by up to ± 0.1 produced only small shifts in mean GMean. In TS1, GMean ranged from 0.53 (LLR=+0.1) to 0.58 (LLR=0.1), a spread of 0.050 (8.6% relative to the highest value), and this shift was consistent and near-monotonic: GMean decreased as the threshold moved from 0.1 to +0.1. In TS2, the spread was of similar absolute size, 0.050 (9.2% relative), but the pattern was not monotonic — LLR=0 (mean 0.545) was the highest-performing threshold, with GMean lower on both sides of it. In TS3, the spread was smaller still, 0.032 (5.9% relative), ranging from 0.50 (LLR=+0.1) to 0.53 (LLR=0.05 and LLR=0.1). Taken together, the classifier's GMean is barely sensitive to threshold perturbation in absolute terms in every test set.

S4. Edge weight reshuffling

To assess the impact of edge weights on StrucNS, original edge weights were reshuffled. The exact pipeline was used to retrain the model and evaluate the performance. GMean dropped across all three test sets (Figure. S9).

S5. Ablation of top SHAP features

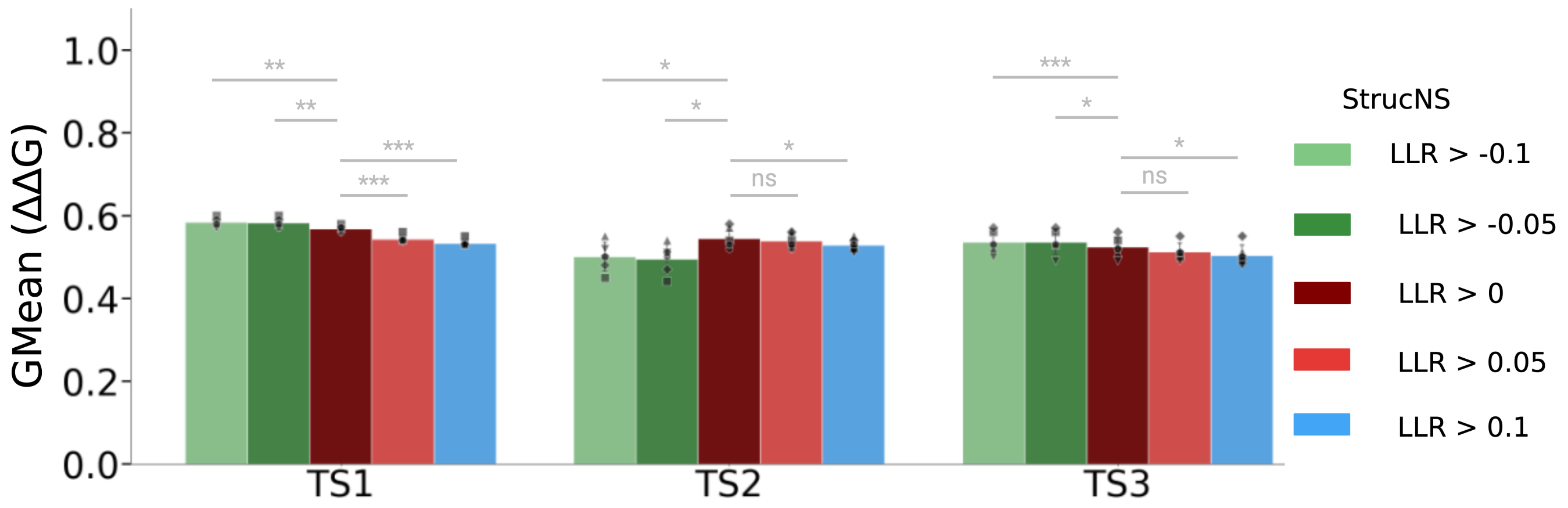

Figure S8 Model performance is not impacted by the LLR threshold. StrucNS tested with the indicated LLR threshold. The Gmean for predicting the stability classification for each test set is reported as the mean ±standard deviation across six train-test splits.

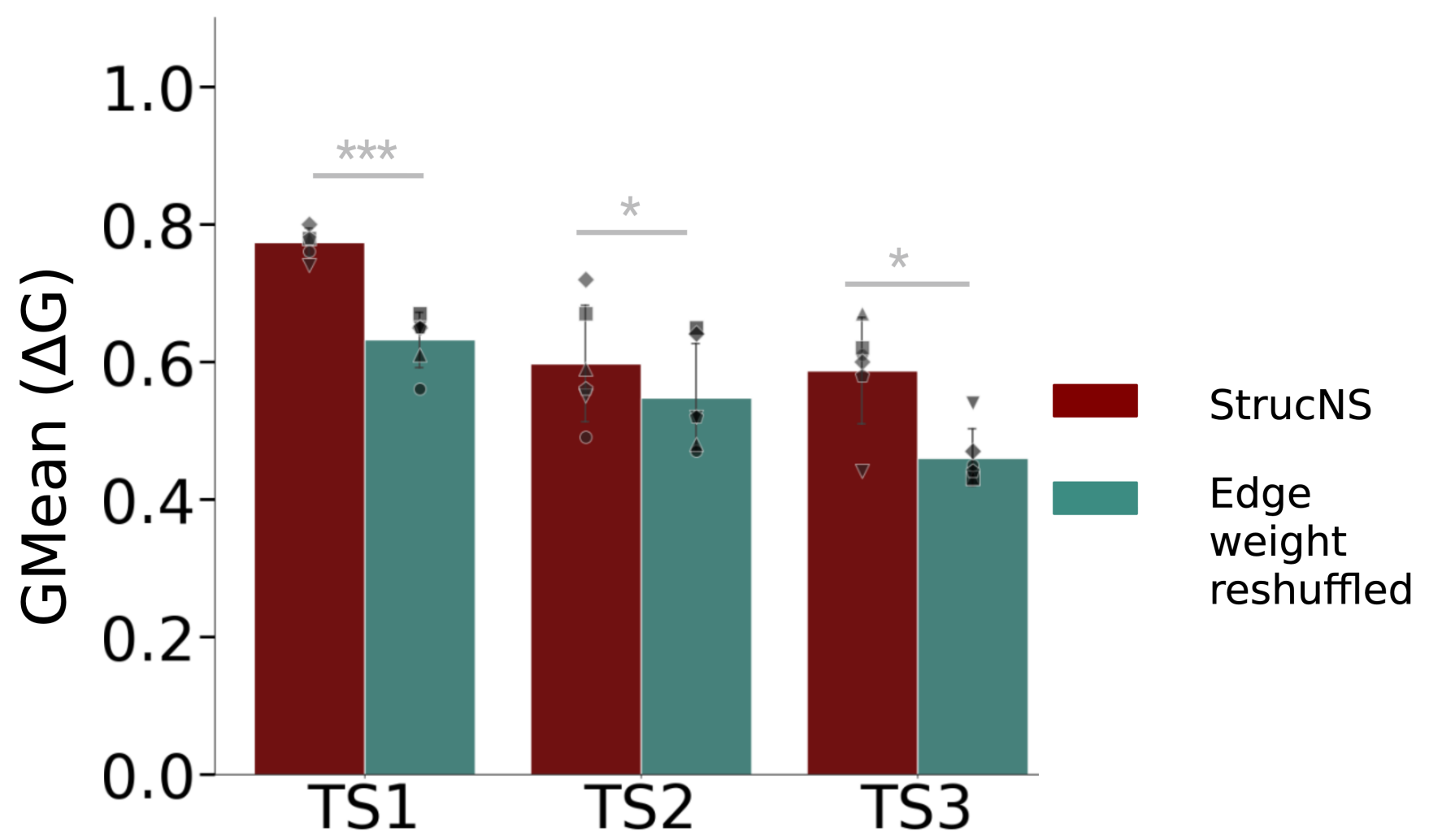

Figure S9 Model performance is impacted by edge weights. StrucNS was trained by reshuffling the edge weights assigned to edges in its base model, then retrained using the same pipeline. Consequently, GMean decreases across all test sets.

To assess the impact of top features on StrucNS, top 10 SHAP features were removed. The exact pipeline was used to retrain the model and evaluate the performance. GMean dropped across TS1 and TS3 (Figure. S10).

S6. Control model implementation

S6.1. ESM-2 Implementation and Evolutionary Scoring

The evolutionary fitness of protein variants was quantified using the Evolutionary Scale Modeling (ESM-2) transformer architecture. We employed the `esm2_t33_650M_UR50D` pre-trained model, consisting of 33 layers and 650 million parameters, implemented via the `esm` (v2.0.0) library in `PyTorch` (v2.0). All inference tasks were executed on NVIDIA A100 GPUs to accelerate the transformer attention mechanism.

Sequence Extraction and Preprocessing

Primary sequences were derived from structural coordinates to ensure parity between structural network analysis and sequence-based scoring. Amino acid sequences were extracted from the ATOM records of PDB files using `Bio.SeqIO.parse` with the `"pdb-atom"` flavor. Sequences exceeding the 1022-residue context window were truncated to meet the transformer's positional encoding constraints.

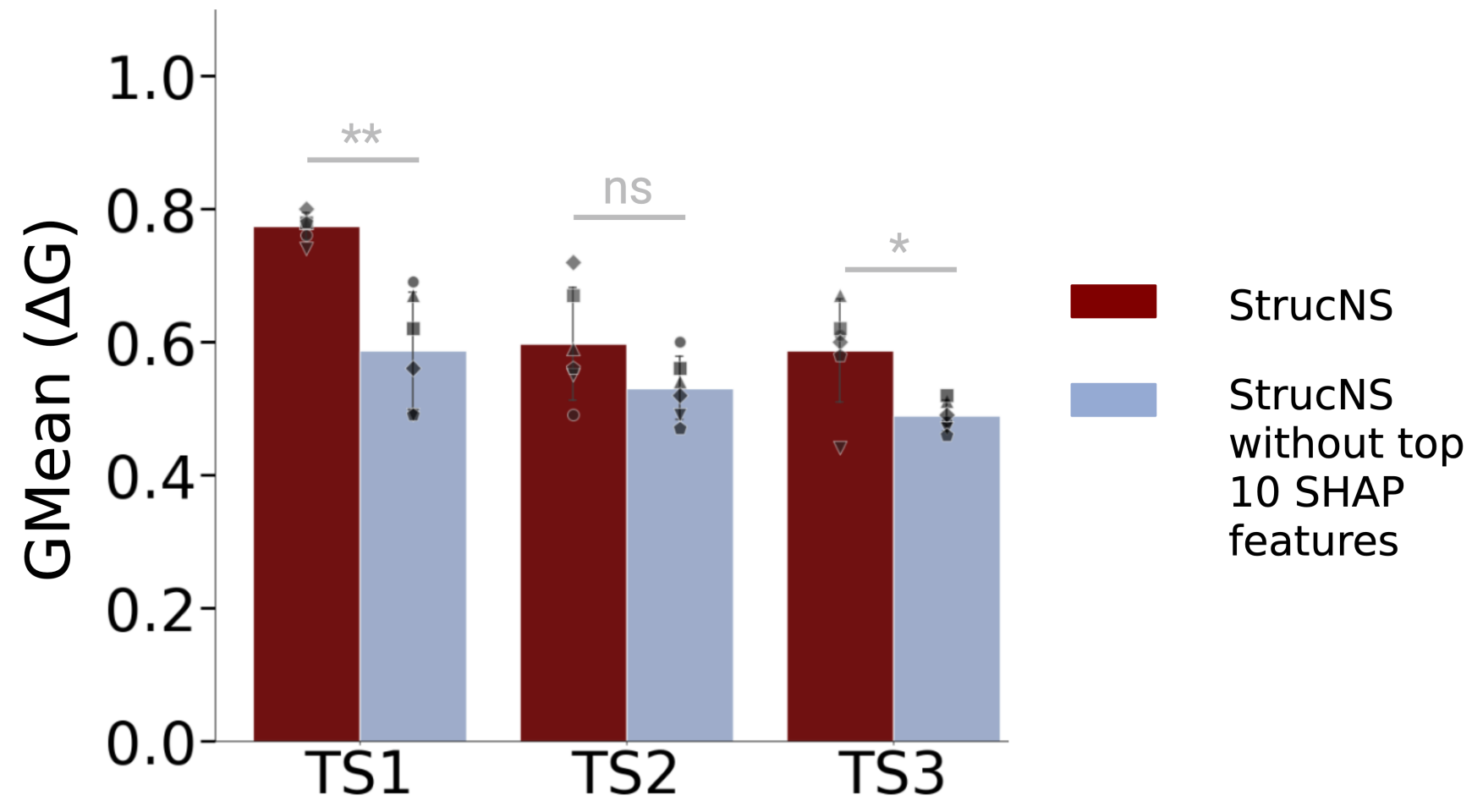

Figure S10 Model performance is impacted by top SHAP features. StrucNS was trained by removing top 10 SHAP features, then retrained using the same pipeline. Consequently, GMean decreases across TS1 and TS3.

Pseudo-Log-Likelihood (PLL) Objective

To evaluate sequence stability, we calculated the pseudo-log-likelihood (PLL) based on the Masked Language Modeling (MLM) objective. For a sequence S, the PLL was computed by iteratively applying the `alphabet.mask_idx` to each position i and summing the log-probabilities of the original amino acid x_i as predicted by the model's logits:

$$PLL(S) = \sum_{i=1}^{L} \log P(x_i \mid S_{\setminus i}; \theta)$$

where $S_{\setminus i}$ represents the sequence with the i-th residue masked, and θ denotes the model parameters. The relative evolutionary impact of a mutation was determined by the Log-Likelihood Ratio (LLR):

$$LLR = PLL(S_{mut}) - PLL(S_{wt})$$

Computational Optimization

The scoring pipeline was optimized for large-scale datasets using a dual-caching strategy. Calculated $PLL(S_{wt})$ values were stored in-memory (`Dict[str, float]`) and persisted to disk via `csv.DictWriter` to eliminate redundant computations for common wild-type backgrounds. The workload was parallelized across high-performance computing clusters using SLURM job arrays, where the `SLURM_ARRAY_TASK_ID` environment variable was mapped to partition-specific CSV inputs for distributed processing.

S6.2. ESM-1v implementation and variant scoring

To establish a baseline for variant effect prediction using zero-shot evolutionary information, we implemented an ensemble of five `ESM-1v` transformer models (`esm1v_t33_650M_UR90S_[1-5]`). Each model in the ensemble consists of 33 layers and 650 million parameters, trained with different random seeds on the UniRef90 database to improve the robustness of the predicted mutational effects.

Ensemble Model Loading and Architecture

The ensemble was instantiated using the `esm.model.esm1.ProteinBertModel` class. Because the models were loaded from raw state dictionaries, we implemented a custom `Namespace` to define the `roberta_large` architecture parameters: 33 layers, an embedding dimension of 1280, 20 attention heads, and a feed-forward network (`ffn_embed_dim`) of 5120. A clean-up logic was applied to the `state_dict` keys to resolve naming conflicts across the five checkpoint iterations, and the language modeling heads (`lm_head`) were dynamically resized to match the target vocabulary.

Wild-type Marginal Scoring Strategy

Variant scoring was executed using the "Wild-type Marginal" strategy, which is computationally efficient for single and multi-point mutations. For each variant, we identified the set of indices $\{pos\}$ where the

mutant sequence (S_{mut}) differed from the wild-type (S_{wt}). The Log-Likelihood Ratio (LLR) was computed by passing the wild-type sequence through the ensemble and comparing the log-probabilities of the mutant and wild-type amino acids at the affected positions:

$$LLR_{ensemble} = \frac{1}{5}\sum_{m=1}^{5}\sum_{i\in\{pos\}}(\log P(x_{i,mut} \mid S_{wt};\theta_m) - \log P(x_{i,wt} \mid S_{wt};\theta_m))$$

where $x_{i,mut}$ and $x_{i,wt}$ are the mutant and wild-type residues at position i, and θ_m represents the parameters of the m-th model in the ensemble. This approach captures the probability of the mutation within the context of the wild-type sequence.

Implementation and Parallelization

Primary sequences were extracted from PDB ATOM records using `Bio.SeqIO` to ensure exact correspondence with the structural models. The inference pipeline was parallelized using SLURM job arrays, with each task processing a distinct partition of the dataset. To ensure reliability during long-running jobs, we implemented a file-stream logic using `csv.DictWriter` and `f.flush()`, allowing for real-time data persistence and the ability to skip previously processed variants upon job resumption.

S6.3. ESM-IF1 Structural Inverse Folding Implementation

To evaluate the structure-sequence compatibility of protein variants, we implemented the `ESM-IF1` inverse folding model (`esm_if1_gvp4_t16_142M_UR50`). This model utilizes a Geometric Vector Perceptron (GVP) Graph Neural Network (GNN) combined with a transformer decoder (142 million parameters) to map 3D structural coordinates to sequence probabilities.

Coordinate Loading and Feature Extraction

Structural inputs were sourced from OmegaFold-predicted PDB files. The model interface utilized the `esm.inverse_folding.util.load_coords` function to extract the backbone coordinates $(N, C\alpha, C)$ of the protein chain. These coordinates serve as the invariant structural backbone upon which the likelihood of both wild-type and mutant sequences was assessed.

Inverse Folding Scoring Objective

The model computes the conditional log-likelihood of a sequence given its 3D backbone coordinates. We quantified the structural stability of each variant by calculating the log-likelihood ($\mathcal{L}$) using the `score_sequence` method. The fitness of a mutation was determined by the change in structural log-likelihood ($\Delta\mathcal{L}_{IF}$) between the mutant structure and the wild-type structure:

$$\Delta\mathcal{L}_{IF} = \mathcal{L}(S_{mut} \mid C_{mut}) - \mathcal{L}(S_{wt} \mid C_{wt})$$

where S represents the amino acid sequence and C represents the 3D atomic coordinates. This metric captures the degree to which a specific mutation is favored or disfavored by the local and global structural environment.

Computational Optimization and Scalability

The implementation featured several performance-enhancing strategies:

- **Dynamic Coordinate Mapping:** An enhanced search logic was implemented in `find_pdb_path` to resolve varied file naming conventions (e.g., `.pdb_wte.pdb` vs. `.pdb.pdb`) across heterogeneous PDB directories.
- **Persistence and Cache Recovery:** To minimize redundant GNN passes, wild-type structural scores were cached in a `wt_score_cache` (`Dict[str, float]`). Upon execution, the script automatically resumed by cross-referencing existing output files to reconstruct the cache and skip completed entries.
- **Inference Monitoring:** Real-time telemetry was implemented to track the average processing time per structural record, providing projected completion windows for large-scale SLURM job arrays.

S6.4. ProteinMPNN Structural Scoring and Evaluation

To assess the structural stability and energetic favorability of variants, we utilized `ProteinMPNN` (v.1.0.1), a graph-based neural network architecture designed for inverse folding. The model evaluates how well a

specific sequence fits a given three-dimensional backbone by calculating the sequence log-likelihood based on structural node and edge features.

Model Execution and Parameterization

The scoring pipeline was implemented via a `subprocess` wrapper in `Python`, calling the `protein_mpnn_run.py` script within a dedicated `Conda` environment. We employed the `v_48_020` model weights, which provide a balance between computational efficiency and accuracy. Scoring was restricted to Chain A (`--pdb_path_chains "A"`) with a `batch_size` of 1 to ensure deterministic score extraction for each specific wild-type and mutant PDB coordinate set.

Log-Likelihood Extraction and Differential Scoring

The `ProteinMPNN` score, representing the average negative log-probability of the sequence given the backbone, was parsed from the resulting FASTA output using regular expression (`re`) matching:

$$Score_{MPNN} = -\frac{1}{L}\sum_{i=1}^{L}\log P(s_i \mid \text{backbone}; \theta)$$

For each variant, we calculated the scores for both the wild-type (LL_{WT}) and mutant (LL_{mut}) structures. The structural impact was quantified as the Log-Likelihood Ratio (LLR_{MPNN}):

$$LLR_{MPNN} = LL_{WT} - LL_{mut}$$

In this convention, a positive LLR indicates that the mutation is less compatible with its structure compared to the wild-type, suggesting a potential loss of stability or fitness.

Automated Pipeline and Data Management

Computational throughput was optimized through several implementation strategies:

- **Dynamic Directory Mapping:** The script utilized a custom `get_mpnn_score` function to handle complex PDB naming conventions, automatically resolving suffixes (e.g., `.pdb_suffix.pdb`) associated with mutant structures.
- **Parallelized Batch Processing:** Distributed execution was managed via SLURM job arrays, where the `SLURM_ARRAY_TASK_ID` was utilized to partition the input datasets into discrete tasks.
- **Resource Cleanup and Persistence:** To avoid filesystem overhead during large-scale scoring, a temporary working directory (`temp_mpnn_[PID]`) was created for each process to store intermediate FASTA outputs. This directory was purged using `shutil.rmtree` upon successful score extraction and row-wise persistence to the output CSV.

S6.5. ThermoMPNN Thermodynamic Stability Prediction

To provide explicit thermodynamic benchmarks, we implemented the `ThermoMPNN` model, which extends the ProteinMPNN architecture with a dedicated stability prediction head. We utilized the `single` configuration of the `v2` model, specifically parameterized to predict $\Delta\Delta G$ values for single-point mutations based on 3D structural environments.

Model Architecture and Featurization

The implementation utilized a hierarchical featurization process via `tied_featurize_mut` from the `thermompnn.datasets` module. Structural data were extracted from OmegaFold-generated PDB files and converted into graph-based features including backbone coordinates (X), amino acid sequences (S), and residue indices. To ensure numerical stability during the forward pass, we applied `torch.nan_to_num` to the coordinate tensors.

Forward Pass and $\Delta\Delta G$ Mapping

Stability predictions were generated by passing the featurized structural graphs through the ProteinMPNN encoder. We extracted the hidden states (`all_mpnn_hid`) from the final model layers and applied a light attention mechanism (`model.light_attention`) to aggregate local structural contexts. The final $\Delta\Delta G$ map was produced by the `model.ddg_out` head. To ensure the predictions were relative to the starting structure, we normalized the output map by subtracting the wild-type stability score:

$$\Delta\Delta G_{map} = \text{Map}_{raw} - \text{batched\_index\_select}(\text{Map}_{raw}, S_{WT})$$

The final prediction for a specific mutation X_iY (where X is the wild-type residue at position i and Y is the mutant) was retrieved by indexing the normalized `ddg_map` at the corresponding position and amino acid identity.

Automated Pipeline for Large-Scale Mutagenesis

The computational pipeline integrated several custom modules to handle high-throughput evaluation:

- **Mutation Parsing:** We developed a regex-based parser (`parse_mutation`) to extract wild-type identity, sequence position, and mutant identity directly from standardized GraphML filenames.
- **Priority-Based PDB Resolution:** The `get_wt_pdb_path` function implemented a multi-directory search logic to resolve wild-type structures across distinct processing tiers (e.g., `omegafold_pdbs_WT` and secondary search paths).
- **Fault-Tolerant Execution:** The script featured a robust error-handling framework that logged specific failure modes (e.g., `IndexError`, `ModelError`, or `WT_NotFound`) directly to the output CSV, ensuring dataset integrity even when structural data were missing or corrupted.

S6.6. Supervised ESM-2 Embedding and Classification

To leverage the high-level semantic information captured by large-scale protein language models, we implemented a supervised learning framework that combines fixed feature extraction from a pre-trained transformer with a downstream deep neural network optimized for stability classification.

ESM-2 Feature Extraction and Mean Pooling

Sequence-level representations were generated using the `esm2_t33_650M_UR50D` architecture. For each sequence, hidden states were extracted from the final transformer layer ($layer = 33$), resulting in a 1280-dimensional vector per residue. To obtain a fixed-length global representation for each protein, we applied a mean-pooling operation across the sequence dimension (excluding `<cls>` and `<eos>` tokens):

$$E = \frac{1}{L}\sum_{i=1}^{L} \mathbf{h}_i^{(33)}$$

where L denotes the sequence length and $\mathbf{h}_i^{(33)}$ represents the hidden representation of residue i. Inference was performed on NVIDIA A100 GPUs using a checkpoint-aware script to ensure data persistence and reproducibility.

Data Preprocessing and Dimensionality Reduction

Prior to training, the 1280-dimensional embeddings were processed through a multi-stage pipeline:

- **Imputation:** Missing values were addressed using a `SimpleImputer` with a mean-value strategy.
- **Standardization:** Features were normalized to zero mean and unit variance using `StandardScaler`.
- **Principal Component Analysis (PCA):** To mitigate the curse of dimensionality and prevent overfitting, we applied PCA, retaining enough components to explain 99% of the total variance.

Hyperparameter Optimization and Model Architecture

The downstream classifier was developed using `TensorFlow/Keras`. The target variable was binarized based on experimental ΔG values from the Tsuboyama et al. dataset, defining stable variants ($y = 1$) as those with $\Delta G > 3$ kcal/mol.

To determine the optimal architecture, we utilized `Optuna` for 300 trials of Bayesian optimization. The search space included the number of hidden layers (1–4), layer widths (32–512 units), dropout rates (0.1–0.5), and the conditional application of Batch Normalization. A family-aware splitting strategy was employed to ensure that proteins from the same evolutionary family were restricted to either the training or validation sets, preventing data leakage.

Training and Evaluation

The final model was retrained using the best hyperparameters (`Adam` optimizer, variable learning rates). We implemented a custom `F1ScoreCallback` to monitor the F1-score at each epoch and restore weights

associated with the highest validation performance:

$$F1 = 2 \cdot \frac{\text{Precision} \cdot \text{Recall}}{\text{Precision} + \text{Recall}}$$

The model was evaluated across three independent test sets, with performance logged via confusion matrices and class-specific recall to assess the model's robustness across diverse protein scaffolds.

S6.7. Supervised UniRep Embedding and Classification Pipeline

To capture the statistical and structural features encoded within protein primary sequences, we implemented a supervised framework utilizing UniRep—a multiplicative Long Short-Term Memory (mLSTM) recurrent neural network—to generate fixed-length vector representations for stability classification.

UniRep Feature Extraction and mLSTM Processing

The UniRep embeddings were generated using the `jax_unirep` framework. Sequences were standardized and filtered to include the 20 standard amino acids and the unknown residue token (`X`). The mLSTM architecture processed each sequence to produce 1900-dimensional hidden states. To obtain a global representation for each variant, a mean-pooling operation was applied:

$$E_{UniRep} = \frac{1}{L} \sum_{i=1}^{L} \mathbf{h}_i$$

where L is the sequence length and $\mathbf{h}_i \in \mathbb{R}^{1900}$. Computational throughput was optimized by parallelizing extraction across high-performance computing clusters using Slurm job arrays to partition the dataset into discrete chunks.

Preprocessing and Dimensionality Reduction

Prior to classification, the 1900-dimensional vectors underwent a robust preprocessing pipeline:

- **Family-Aware Stratification:** Variants were partitioned into training and validation sets using family-level metadata to prevent data leakage and ensure evolutionary independence between splits.
- **Standardization and Imputation:** Features were centered and scaled using `StandardScaler`, with mean-value imputation applied to any missing data points.
- **PCA Projection:** Principal Component Analysis (PCA) was utilized to reduce dimensionality while retaining 99% of the cumulative explained variance.

Supervised Classification and Optimization

A deep multi-layer perceptron (MLP) was trained to predict protein stability as a binary classification task ($\Delta G > 3$ kcal/mol). We utilized `Optuna` for 300 trials of Bayesian hyperparameter optimization, tuning architecture depth, layer widths, dropout rates, and learning schedules. The final model weights were selected based on the peak validation F1-score:

$$F1 = 2 \cdot \frac{\text{Precision} \cdot \text{Recall}}{\text{Precision} + \text{Recall}}$$

Performance was rigorously evaluated on independent test sets, utilizing confusion matrices and class-specific recall to validate the model's generalizability across diverse protein families.

S7. Ablation models

S7.1. Topological Null Model: Random Edge Rewiring

To isolate the contribution of protein network topology from simple spatial proximity, we generated randomized graphs using a Topology Noising strategy. This method produces a null model where the protein fold and edge weight distribution are conserved, but the specific biological connectivity is destroyed.

S7.1.1. Conservation of Graph Invariants

The randomization process was constrained to maintain the first-order properties of the native protein graph $G = (V, E)$, where V represents the set of nodes (amino acid residues) and E represents the

set of edges (physicochemical interactions). For each input graph, we preserved the node set V and its associated spatial attributes (PDB Cartesian coordinates). We extracted the set of original edge weights W, where each weight $w \in W$ corresponds to the strength of a specific bond (e.g., hydrogen, peptide, or hydrophobic) as defined by the physicochemical properties of the residues.

S7.1.2. Algorithmic Implementation of Random Rewiring

The original edge set E was entirely purged, effectively removing the biological connectivity while leaving the residues fixed in 3D space. A new, stochastic edge set E_{rand} was then populated using a batch-sampling approach to achieve an identical edge count $|E_{rand}| = |E|$.

The generation of E_{rand} followed a uniform sampling distribution:

- **Node Selection:** Pairs of node indices (u, v) were sampled from a discrete uniform distribution $U(0, |V| - 1)$, where $|V|$ is the total number of residues in the protein.
- **Uniqueness Constraints:** To ensure an undirected, simple graph, self-loops ($u = v$) were prohibited. Pairs were sorted such that $(u, v) \equiv (v, u)$ to prevent duplicate edges.
- **Batch Processing:** Sampling was performed in batches of $1.5 \times |E|$ to optimize computational efficiency against collisions, continuing until the target edge cardinality was reached.

S7.1.3. Weight Shuffling and Mapping

To preserve the exact chemical "budget" of the protein while removing structural context, the weight vector W was permuted using a Fisher-Yates shuffle. Each shuffled weight $w'_k \in W_{shuffled}$ was then assigned to a corresponding edge $e_k \in E_{rand}$.

The resulting adjacency matrix A_{rand} for the noised graph is defined as:

$$A_{ij} = \begin{cases} w'_{ij} & \text{if } (i, j) \in E_{rand} \\ 0 & \text{otherwise} \end{cases}$$

Where w'_{ij} is the randomized weight assigned to the connection between residues i and j. This ensures that the total graph weight $\sum w_{ij}$ and the distribution of bond strengths are invariant, but the adjacency relationship between residues is entirely decoupled from their sequence distance or biological proximity. To ensure the reproducibility of this stochastic topology across different job arrays, a global random seed of 42 was applied to the randomization modules.

S7.2. Geometric Null Model: Rigid Body Random Walk

To assess the impact of the native 3D fold on network topology, we implemented a **Geometric Random Walk** protocol. This approach generates a non-biological, "random coil" spatial configuration of the protein while preserving the local rigid-body geometry of individual amino acids and their primary sequence order.

S7.2.1. Stochastic Backbone Trajectory

The global trajectory of the protein backbone was modeled as a self-avoiding-like random walk in $\mathbb{R}^3$. Let $\mathbf{p}_i$ denote the Cartesian coordinate vector (x, y, z) of the α-carbon ($C\alpha$) for the i-th residue in a sequence of total length N. The coordinates were generated iteratively according to the following process:

1. **Initialization:** The first residue in the sequence was anchored at the origin, where $\mathbf{p}_1 = (0, 0, 0)$.
2. **Directional Sampling:** For each subsequent residue $i \in \{2, \ldots, N\}$, a random unit direction vector $\hat{\mathbf{d}}_i$ was sampled from a uniform distribution on a sphere. This was achieved via Gaussian normalization:

$$\hat{\mathbf{d}}_i = \frac{\mathbf{v}}{\|\mathbf{v}\|}, \quad \mathbf{v} \sim \mathcal{N}(0, 1)^3$$

   where $\mathbf{v}$ is a 3D vector of independent standard normal samples.
3. **Step Displacement:** To simulate backbone flexibility, a scalar step size s_i was sampled from a uniform distribution $U(2.0, 4.0)$ Å. The position of the current residue was then defined as:

$$\mathbf{p}_i = \mathbf{p}_{i-1} + (s_i \cdot \hat{\mathbf{d}}_i)$$

   where $\mathbf{p}_{i-1}$ is the position of the preceding residue in the sequence.

S7.2.2. Rigid-Body Side-Chain Transformation

To maintain the chemical integrity of individual residues, each amino acid was treated as a rigid body. Let $\{\mathbf{r}_{i,j}\}$ be the set of relative coordinate vectors for all atoms j in residue i, defined as the distance from the native $C\alpha$ position in the original PDB structure. For each residue i, a unique random rotation matrix $\mathbf{R}_i \in SO(3)$ was generated. The final transformed absolute coordinates $\mathbf{x}_{i,j}$ for every atom j in the null model were calculated as:

$$\mathbf{x}_{i,j} = \mathbf{p}_i + (\mathbf{R}_i \cdot \mathbf{r}_{i,j})$$

In this equation, $\mathbf{p}_i$ represents the new $C\alpha$ position from the random walk, and $\mathbf{R}_i \cdot \mathbf{r}_{i,j}$ represents the randomly reoriented side-chain atoms. This ensures that internal bond lengths and angles within each residue remain identical to the biological source, while their orientation relative to the chain and the global fold is randomized.

S7.2.3. Geometry-Dependent Edge Re-calculation

Following the spatial randomization, the network edges E_{rw} were re-calculated *de novo* based on the new coordinate set $\mathbf{x}_{i,j}$. Unlike the topological noising where edges were sampled stochastically, here the edges are a deterministic function of the new geometry:

- **Sequence Edges:** Peptide bonds were maintained between adjacent residues $(i, i+1)$ in the sequence.
- **Non-Covalent Interactions:** Edge types such as hydrogen bonds, hydrophobic clusters, and disulfide bridges were assigned to the new edge set E_{rw} only if the transformed atomic coordinates $\mathbf{x}_{i,j}$ met the specific distance thresholds d defined in the native interaction model (e.g., $d < 3.5$ Å for hydrogen bonds).

The resulting graph $G_{rw} = (V, E_{rw})$, where V is the set of residues and E_{rw} is the new set of geometry-derived edges, represents a chemical polymer with the same composition as the native protein but lacking an evolved tertiary structure. Reproducibility was ensured by applying a global random seed of 42 to the `numpy.random` and `scipy.spatial.transform` modules.

S7.3. Geometric Null Model: Bounding Box Randomization (Random box model)

To evaluate the significance of the spatial distribution without the influence of sequence connectivity, we implemented a **Bounding Box Randomization** strategy. This model creates a gas-like distribution of residues that preserves the overall volume and density of the native protein but eliminates all structural and sequential context.

S7.3.1. Preservation of Global Spatial Envelope

The randomization is constrained by the original spatial dimensions of the native protein to ensure the null model occupies a comparable volume. For a protein with N residues, the original Cartesian coordinates (x_i, y_i, z_i) were used to define a bounding box:

$$\begin{aligned} x_{min}, x_{max} &= \min(x_{1\ldots N}), \max(x_{1\ldots N}) \\ y_{min}, y_{max} &= \min(y_{1\ldots N}), \max(y_{1\ldots N}) \\ z_{min}, z_{max} &= \min(z_{1\ldots N}), \max(z_{1\ldots N}) \end{aligned}$$

These limits define the domain $\Omega = [x_{min}, x_{max}] \times [y_{min}, y_{max}] \times [z_{min}, z_{max}]$, ensuring that the randomized residues are contained within the same macroscopic 3D envelope as the biological structure.

S7.3.2. Uniform Spatial Sampling

Each residue $i \in \{1, \ldots, N\}$ is assigned a new position $\mathbf{p}'_i$ sampled from a continuous uniform distribution across the three spatial axes:

$$\begin{aligned} x'_i &\sim U(x_{min}, x_{max}) \\ y'_i &\sim U(y_{min}, y_{max}) \\ z'_i &\sim U(z_{min}, z_{max}) \end{aligned}$$

Unlike the random walk model, the distance between residue i and $i+1$ is not constrained, effectively shattering the polypeptide chain into a set of independent points in space. This isolates the effect of residue density and chemical composition from the effects of backbone topology.

S7.3.3. Graph Reconstruction and Feature Extraction

In this null state, the "PDB position" attribute (pos_i) is updated to the randomized coordinates:

$$\text{pos}_i = (x'_i, y'_i, z'_i)$$

This updated position serves as the basis for all downstream spatial feature calculations.

S8. SHAP Analysis

To interpret the contributions of individual features to the predictive performance of the deep learning models, we utilized **SHAP (SHapley Additive exPlanations)**. SHAP is a game-theoretic approach that assigns each feature an importance value (a Shapley value) for a particular prediction, representing the change in the expected model output when that feature is observed versus when it is missing.

Given the computational complexity of the deep learning architectures (Cases 1–6), we employed the `KernelExplainer` with a K-means summary of the training data (20 centroids) used as the background distribution. This allowed for an efficient estimation of feature impact across a unified test set ($N \approx 1200$ samples across all cases).

S8.1. Quantitative Metrics for Feature Importance

For each feature i, the primary metric of importance is the **Mean Absolute SHAP value**, defined as:

$$I_i = \frac{1}{N} \sum_{j=1}^{N} |\phi_i^{(j)}| \tag{S6}$$

Where $\phi_i^{(j)}$ is the SHAP value for feature i in sample j. Based on this, we derived two secondary metrics to evaluate the contribution of different feature engineering categories:

1. **Importance Share (%)**: The cumulative impact of a specific category C relative to the total model signal. This indicates the total "weight" assigned to a specific pipeline stage.

$$\text{Importance Share}_C = \left(\frac{\sum_{i \in C} I_i}{\sum_{all} I_i} \right) \times 100 \tag{S7}$$

2. **Average Potency**: The mean importance of features within a category, used to identify which stages provide the highest quality signal per individual feature (also referred to as average importance per feature).

$$\text{Average Potency}_C = \frac{\sum_{i \in C} I_i}{N_C} \tag{S8}$$

Where N_C is the total number of features contained within category C.

S8.2. Categorization Framework

To analyze the biological and structural logic of the model, the 294 features were bifurcated through three distinct categorization lenses:

- **Pipeline Stage**: Features were grouped by their mathematical origin into *Connectivity-based* (topology), *Geometry-based* (spatial metrics), and *Projection-based* (centroid/volume metrics).
- **Multi-Resolution Subnetworks**: Features were grouped by their subnetwork index ($\_0$ to $\_5$). This identifies which structural resolution threshold contains the most critical predictive information.
- **Intra- vs. Inter-Subnetwork**: Features were classified as *Intra-subnetwork* (representing local topological slices) or *Inter-subnetwork* (representing global chemical interactions such as Hydrophobic-Polar pairings).

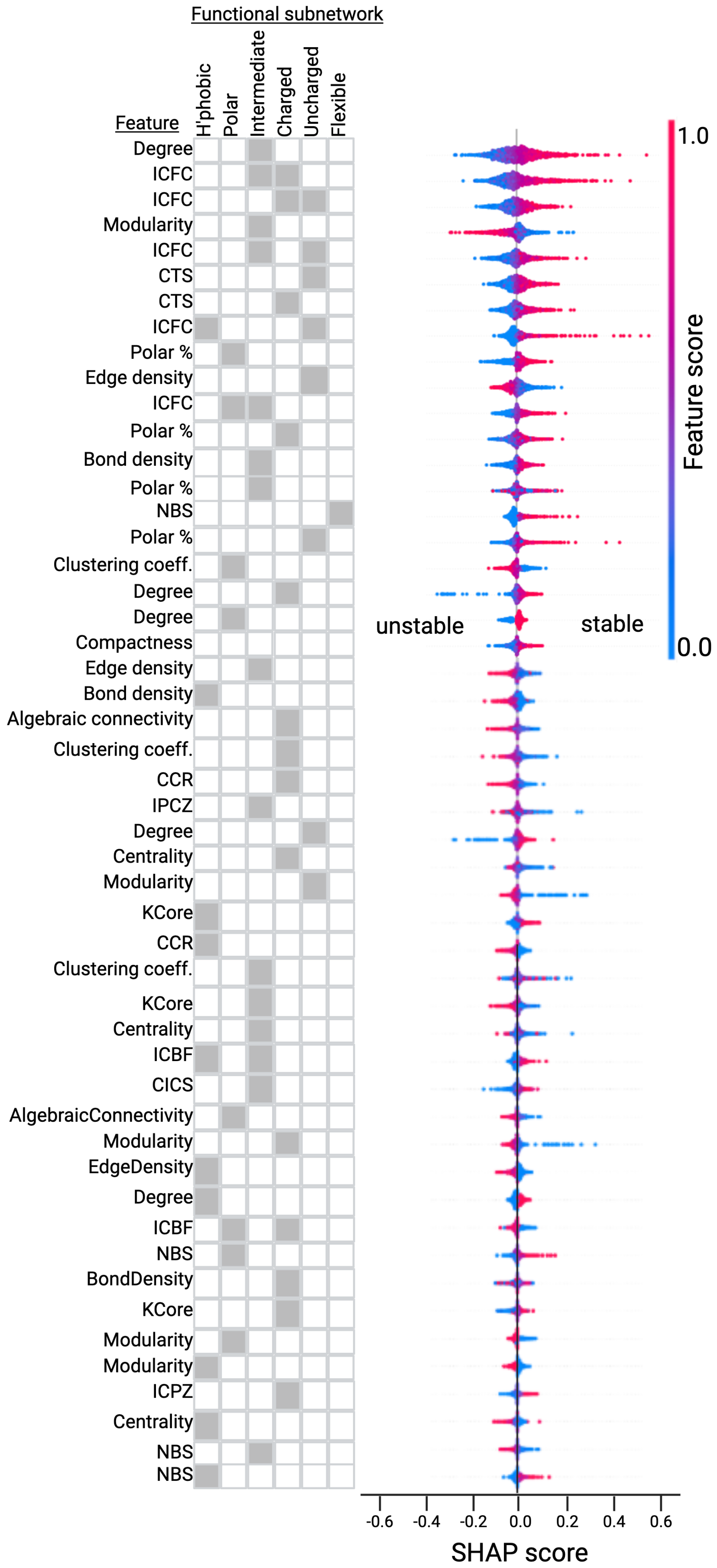

Figure S11 Individual topological features are ranked by their impact on model predictions using SHAP. Top 50 features and their impact is shown.
